# Spores Among Feathers: Evaluating Fungal Diversity in Taxidermized Birds and the Discovery of a New Species

**DOI:** 10.3390/microorganisms14091894

**Published:** 2026-08-26

**Authors:** Luís Fernandes, Diana S. Paiva, Emília Pereira, Célia Cabral, Nuno Mesquita, António Portugal

**Affiliations:** 1 Centre for Functional Ecology (CFE)—Science for People & the Planet, Department of Life Sciences, University of Coimbra, Calçada Martim de Freitas, 3000-456 Coimbra, Portugal; luis.dsfernandes@hotmail.com (L.F.); emiliamaria1999@gmail.com (E.P.); celia.cabral@fmed.uc.pt (C.C.); inunomesquita@gmail.com (N.M.); aportuga@bot.uc.pt (A.P.); 2 Centre for Innovative Biomedicine and Biotechnology (CIBB), University of Coimbra, 3000-548 Coimbra, Portugal; 3 Coimbra Institute for Clinical and Biomedical Research (iCBR), Clinic Academic Centre of Coimbra (CACC), Faculty of Medicine, University of Coimbra, 3000-548 Coimbra, Portugal; 4 Instituto de Histologia e Embriologia, Faculty of Medicine, University of Coimbra, Rua Larga, Edifício da FMUC, Pólo 1, 2º piso, 3004-504 Coimbra, Portugal; 5 TERRA—Associate Laboratory for Sustainable Land Use and Ecosystem Services, Department of Life Sciences, University of Coimbra, Calçada Martim de Freitas, 3000-456 Coimbra, Portugal; 6 FitoLab—Laboratory for Phytopathology, Instituto Pedro Nunes, Rua Pedro Nunes, 3030-199 Coimbra, Portugal

**Keywords:** cultural heritage, biodeterioration, fungal contamination, museum collections, new taxon, *Periconia*, fungal phylogeny, fungal taxonomy

## Abstract

Fungal organisms play a major role in the contamination and biodeterioration of natural history museum collections and cultural heritage as a whole, leading to the potential loss of key knowledge preserved by these. As part of the ongoing efforts to study the fungal contaminations observed in the Science Museum of the University of Coimbra (Portugal) collections, eight taxidermized birds were sampled and 27 fungal isolates were retrieved. A comprehensive analysis of these isolates led to the identification of 14 different species belonging to 13 different genera, with *Aspergillus* accounting for 37% (1 species), *Talaromyces* for 11.11% (1 species); *Cladosporium*, *Paramicrodochium*, and *Periconia* accounting for 7.4% each (2, 1, and 1 species, respectively); and *Arcopilus*, *Beauveria*, *Coprinellus*, *Hyphodermella*, *Mycoaciella*, *Neokalmusia*, *Paraeutypella*, and *Penicillium* collectively accounting for 3.7% each, all represented by a single species. Among these were two isolates of an unknown *Periconia* species. These isolates were thoroughly studied through an integrative analysis based on a multi-locus phylogeny of a combined dataset of ITS rDNA, LSU, SSU, *TEF1α*, and *RPB2*, along with morphological characteristics. Based on the data obtained from this study, we propose a new addition to this genus, *Periconia callaina* sp. nov. This discovery provides further insight into the fungal diversity present in natural history museums and the communities that colonize and threaten our cultural heritage.

## 1. Introduction

Natural History Museums accommodate important repositories of cultural heritage from fields as diverse as minerology, anthropology and zoology. Zoology collections are composed of a variety of animal specimens preserved via taxidermy, skeleton preparation and other forms, and can be used both as datasets for biodiversity studies [1,2], as well as genetic material repositories for studies targeting genomics, paleogenomics and paleoproteomics to provide valuable insights into the evolutionary history of the species represented in these collections [2,3]. With an estimate of 3 billion specimens housed in natural history collections worldwide, these have been instrumental in demonstrating the effects of climate change and human activity in animal populations by comparing modern specimens to those present in these collections [4]. As such, the ever-present threat of biodeterioration that all forms of cultural heritage face is of particular concern in this context.

While biodeterioration can be caused by the activity of a wide range of living organisms, including, but not limited to, archaea, algae, bacteria and fungi, that through chemical and/or mechanical processes affect the appearance and the integrity of the materials [5], in the context of museums and historic collections such as those found in Natural History Museums, insects and fungi pose the greatest threats [6].

Fungi in particular are a major concern, due to their ubiquitous nature, complex and varied metabolic pathways and ability to thrive even in the most extreme environments [7]. These characteristics allow fungi to colonize and deteriorate the various materials that make up historically relevant artifacts [8]. This colonization often leads to physical and chemical deterioration processes on the surface and/or on the interior of the colonized object, resulting in significant aesthetic and structural damage [9,10]. Chemical processes are often associated with the colonizer’s metabolism, both by substrate digestion and by the excretion of potentially damaging metabolites to the contaminated area. Excretion of metabolites and enzymes such as cellulases, proteases and keratinases can lead to material dissolution, the formation of soluble or insoluble salts and the deterioration of artifacts [11,12,13]. While a larger number of studies has provided insight into the role of fungi in the deterioration of stone monuments [10,14,15,16,17], books and parchments [11,18,19], and wooden artefacts [20,21], fungal based deterioration of zoology specimens in Natural History Museums is largely unexplored. Nevertheless, some work has been conducted in this field already. Pinzari and colleagues have conducted extensive research regarding the fungal contamination of bone and demonstrated that this substrate provides a niche for specialized fungi in museum environments [7]. *Aspergillus*, *Chaetomium* and *Mucor* species are often associated with Natural History collections and were found to be responsible for the contamination of taxidermized bat specimens and pinned entomological collections [22]. These genera are commonly associated with museum environments alongside *Alternaria*, *Cladosporium* and *Penicillium* [8]. In addition to their potential for causing biodeterioration, the presence of these fungi in museum environments may also pose health risks to staff and visitors, with genera such as *Aspergillus* and *Penicillium* being recognized for their potential to cause respiratory infections and other adverse health effects [23].

The Science Museum of the University of Coimbra (MCUC), part of the “Universidade de Coimbra, Alta e Sofia” UNESCO World Heritage Site, houses a collection of over 650,000 artifacts and objects of cultural significance, with a strong emphasis on preserved animal specimens, and is currently facing severe issues related to fungal contamination of its collections [24]. As part of the ongoing efforts to understand, characterize and control fungal populations threatening the MCUC collections, a set of taxidermized birds showing clear signs of contamination was selected for sampling and study.

Thus, the goal of our work was to isolate and characterize fungal contaminants present in the specimens, as well as determining the taxonomic relationships of two identical isolates obtained during the course of this study. Multi-locus phylogenetic and morphological analyses showed that these isolates represent an undescribed species belonging to the *Periconia* genus (*Periconiaceae*) that is taxonomically resolved here.

## 2. Materials and Methods

### 2.1. Site Description and Samples Collection

**Figure 1:**
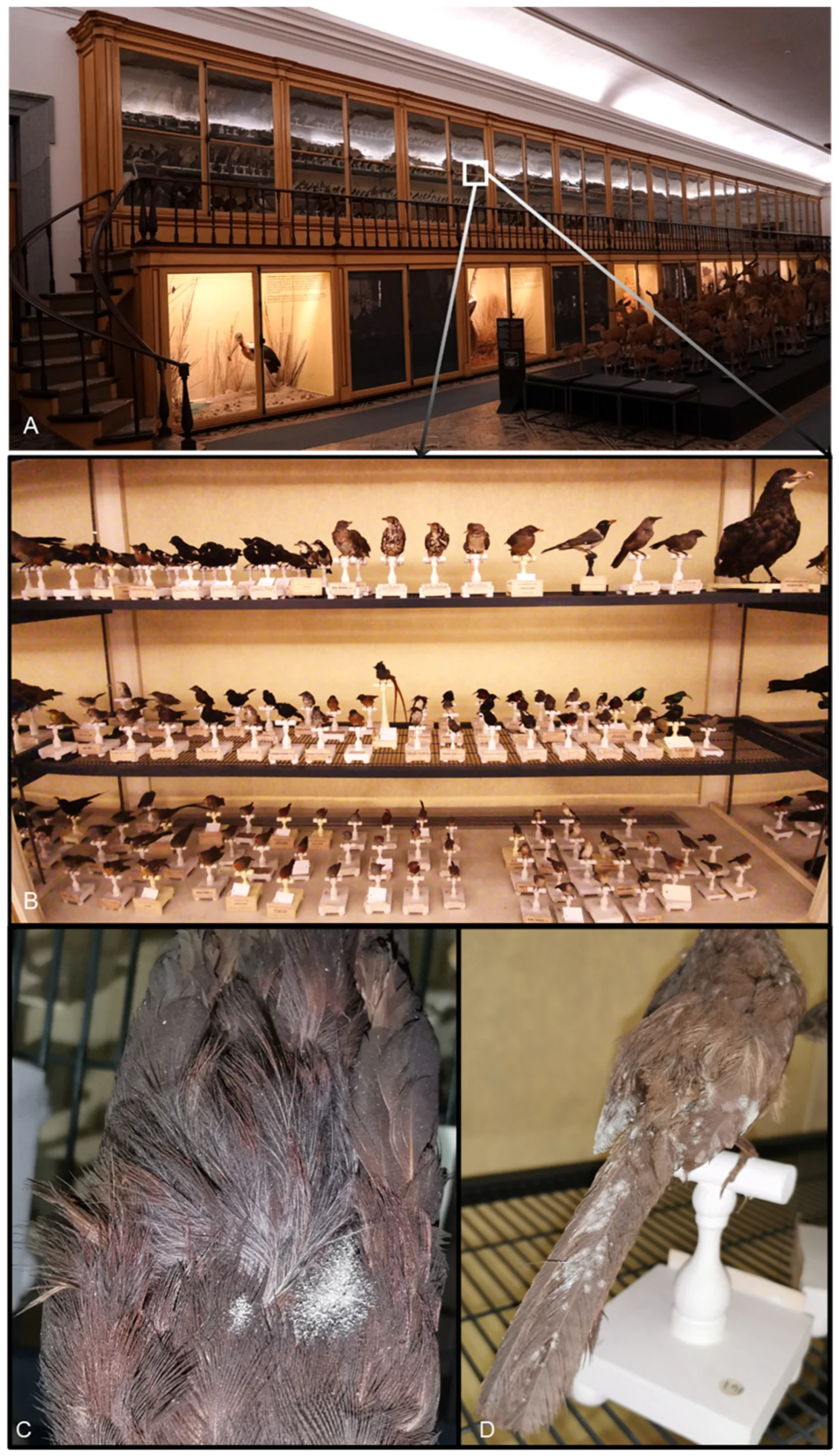
Science Museum of the University of Coimbra. (**A**). Overview of the exhibition room displaying taxidermized birds from the zoological collection. (**B**). Close-up of the taxidermized bird collection. (**C**,**D**). Close-up of two of the sampled specimens showing visible fungal growth.

Currently undergoing a severe reform, contending with limited resources for conservation, and facing challenges in maintaining optimal environmental conditions, the MCUC finds itself with most of its collections closed to the public, stored in archives or closed rooms. All this has led to outbreaks of fungal contamination in different collections, rooms and archives. One such outbreak was observed in a room currently closed to the public, containing mostly taxidermized animals, primarily in a long cabinet with hundreds of taxidermized birds (Figure 1). Eight bird specimens of the African Birds collection displaying macroscopically visible fungal growth were selected for sampling. They represented *Turdoides jardineii tamalakanei* Meyer de Schauensee, R 1932 (ZOO.0038791, ZOO.0038792); *Nectarinia bocagii* Shelley, GE 1879 (ZOO.0000395); *Chalcomitra senegalensis saturatior* Reichenow, A 1891 (ZOO.0039018, ZOO.0039012, ZOO.AVE.AFR.0182.05); and *Melaenornis pammelaina pammelaina* Stanley, ES 1814 subspecies (ZOO.0038991, ZOO.0038992). All specimens originate from the Benguela region in Angola, dating back to the late 19th century.

Sampling was performed using non-invasive methods by swabbing sterile nitrocellulose membranes on areas showing clear evidence of fungal growth, under semi-aseptic conditions. The obtained samples were kept in sealed, otherwise empty and sterile Petri plates and stored in the dark at room temperature and were processed within 6 h from the time of collection. A digital thermohygrometer was used to measure the temperature (T) and relative humidity (RH) at the beginning and the end of the sampling procedure. The average values were T 18 °C and RH 71%.

Given the historical and fragile nature of the specimens, sampling was designed to minimise physical disturbance while targeting areas with visible evidence of fungal growth. Sampling was therefore restricted to accessible, visibly affected areas, which may not fully represent the overall fungal diversity associated with each specimen. Furthermore, the heterogeneous nature of taxidermized specimens and the uneven distribution of fungal growth across their surfaces were considered when selecting sampling sites.

### 2.2. Culturable Fungal Isolation, Identification and Analysis

Each nitrocellulose membrane used for sampling was washed with 2 mL of sterile 0.9% (*w*/*v*) NaCl solution, and the resulting suspension was collected. Per resulting sample, aliquots of 100 µL were plated on Potato Dextrose Agar (PDA; Difco, Sparks, MD, USA); Malt Extract Agar (MEA; Difco, Sparks, MD, USA); Dichloran-glycerol Agar (DG-18; Oxoid, Basingstoke, UK); and Blood Agar Base (BA, Sigma-Aldrich, St. Louis, MO, USA), in 5 replicates. Streptomycin was added to all media at a concentration of 0.5 gL^−1^ to prevent bacterial growth. Inoculated media plates were incubated at 25 ± 2 °C aerobically and left in the dark for 3 months. All emerging colonies in each culture medium with significantly different morphology were further isolated to axenic cultures onto both PDA and the original culture medium they were isolated from and incubated at 25 ± 2 °C aerobically. When significant mycelium growth was observed, DNA extraction of the pure cultures was performed with the REDextract-N.AmpTM Plant PCR Kit (Sigma Aldrich, St. Louis, MO, USA), with several modifications, as described by Paiva et al. [15]. DNA obtained was used for PCR amplification of the ITS, LSU, SSU, *RPB2*, *BTUB*, *CAM* and *TEF1α* regions, using the ITS1-F/ITS4 [25,26], LSU1-Fd/LR5 [27,28,29], NS1/NS4 [26], RPB2-5f/RPB2-7cR [30,31], Bt2a/Bt2b [32], CMD5/CMD6 [33], and Tef1-1018F/Tef1-1620R [34] primer pairs, respectively. An initial molecular analysis based on ITS sequencing was performed for all isolates. Based on the preliminary ITS results, additional loci were selected according to established literature for the respective genera, considering commonly used barcoding regions as well as markers demonstrated to provide phylogenetic resolution and support species identification and delimitation. Accordingly, LSU was amplified for species of *Neokalmusia*, *Cladosporium*, *Talaromyces*, *Penicillium*, *Arcopilus*, *Coprinellus*, *Paraeutypella* and *Mycoaciela*; *SSU* for *Neokalmusia*, *Cladosporium*, *Hyphodermella* and *Paraeutypella*; *BTUB* for *Aspergillus* and *Penicillium*; *RPB2* for *Beauveria*; *CAM* for *Aspergillus*; and *TEF1α* for *Paramicrodochium*. PCR reactions, consisting of 12.5 µL of NZYTaq Green Master Mix (NZYTech^TM^, Lisboa, Portugal), 1 μL of each primer (10 mM), 9.5 μL of ultra-pure water and 1 μL of template DNA, for a final amplification volume of 25 μL, were performed using an ABI GeneAmp^TM^ 9700 PCR System (Applied Biosystems, Waltham, MA, USA). Amplification conditions followed Crous et al. and Hunter et al. [35,36] for ITS, LSU, and *TEF1α*, and Houbraken and Samson [37] for SSU, *RPB2* and *CAM*, while *BTUB* followed Lee et al. [38]. Confirmation of the amplification of the ITS, LSU, SSU, *RPB2*, *BTUB*, *CAM* and *TEF1α* regions was conducted through agarose gel electrophoresis (1.2%) stained with GreenSafe Premium (NZYTech™, Lisboa, Portugal) and visualized in a Molecular Imager Bio-Rad Gel Doc XR™ (Bio–Rad, Hercules, CA, USA). Obtained amplicons were purified and sequenced using an ABI 3730xl DNA Analyzer system (96 capillary instruments) at STABVIDA, Caparica, Portugal. Obtained DNA sequences were analysed using the BioEdit Sequence Alignment Editor© v.7.2.5.0 “(https://bioedit.software.informer.com/download/ (accessed on 10 October 2024)” and deposited in the GenBank database under the accession numbers listed in Supplementary Table S1.

Similarity searches were performed using the National Center for Biotechnology Information nucleotide database (NCBI’s) online Basic Local Alignment Search Tool (BLASTNv.2.6), using the highly similar sequences (MEGABLAST) algorithm of BLAST [39]. These results were further confirmed by molecular analysis using the Mycobank online database [40,41], using the Molecular ID Pairwise Alignment tool (https://www.mycobank.org/page/Pairwise_alignment (accessed on 10 February 2025)) with the default parameters and all databases selected. Molecular results were further confirmed with macroscopic and microscopic analysis of taxonomic traits. Isolated strains were identified according to current names listed on Index Fungorum (www.indexfungorum.org) and Mycobank (https://www.mycobank.org/) (accessed on 10 February 2025) [40,41].

The re-isolation of the same species can occur from different samples. Therefore, fungal diversity was analysed according to species presence–absence in each of the eight sampled specimens. A simple co-occurrence network between species in the samples was constructed and visualized in the Cytoscape software (v.3.10.3, http://www.cytoscape.org (accessed on 5 September 2025)) [42].

Following this survey on culturable fungal diversity, isolates that could not be identified at the species level due to low match similarity or inconclusive results, as well as unique morphological characteristics, were recognized as potential new species and maintained in axenic cultures until further morphological and molecular studies were conducted.

### 2.3. Morphological Characterization

Two isolates, which were identical but did not yield a conclusive match in GenBank and were therefore considered potential representatives of novel species, were selected for detailed morphological characterization. The isolates were cultivated on Potato Dextrose Agar (PDA; Difco, Sparks, MD, USA), Malt Extract Agar (MEA; Difco, Sparks, MD, USA), and Oatmeal Agar (OA) for three weeks at 25 °C in darkness. PDA was used to assess general colony growth and morphology, MEA to further evaluate colony characteristics and pigmentation, and OA to promote sporulation and facilitate the observation of reproductive structures and other diagnostic morphological features. Oatmeal Agar was prepared by boiling 30 g of oatmeal and 15 g of agar in 1 L of distilled water prior to sterilization. Inoculation was performed on 9 cm Petri dishes using a three-point pattern. After incubation, colony diameter (longest axis), degree of sporulation, colony colour on both obverse and reverse sides, texture, form, and the release of soluble pigments into the medium were documented through direct observation. Colour descriptions follow the ISCC-NBS Colour System [43]. Macro photographs were taken with a Sony α6100 camera equipped with a Sony f/3.5-5.6 18-135 mm lens.

Micromorphological features were examined directly on growing colonies or using the slide culture technique, prepared from 3- to 5-week-old colonies on Synthetic Nutrient-Poor Agar (SNA) and OA [44]. Observations were made with a Leica DM750 light microscope (Leica, Wetzlar, Germany) equipped with a Leica ICC50W digital camera (Leica, Wetzlar, Germany). Size data were recorded based on at least 30 measurements per structure, and mean dimensions were calculated. Photo plates were assembled in Microsoft PowerPoint 2016.

A holotype (dried specimen) and additional cultures were deposited in the Micoteca da Universidade do Minho (MUM), Braga, Portugal. The new descriptions and nomenclature were registered in MycoBank [40,41].

### 2.4. Molecular Characterization and Phylogenetic Analyses

Following the initial molecular screening and subsequent morphological characterization, a detailed molecular analysis was performed for the two isolates considered potential representatives of novel species. Genomic DNA was extracted from 3-week-old pure cultures following the same procedure described in Section 2.2. The extracted DNA was used to amplify the ITS, LSU, SSU, *RPB2*, and *TEF1α* regions using the primer pairs and amplification conditions described above. These loci were selected based on phylogenetic studies of Periconia, including those published by Phookamsak et al. [45], Liao et al. [46], Su et al. [47], Yang et al. [48], Tian et al. [49], Chen et al. [50], and Pommer et al. [51], in which these markers were shown to be informative for species-level identification and delimitation within the genus. PCR reactions were performed in 50 µL volumes containing 25 µL NZYTaq Green Master Mix (NZYTech, Lisboa, Portugal), 2 µL of each primer (10 mM), 19 µL ultra-pure water, and 2 µL template DNA. Reactions were carried out in an ABI GeneAmp 9700 PCR System (Applied Biosystems, USA). Amplification success was verified by agarose gel electrophoresis (1.2%) stained with GreenSafe Premium (NZYTech, Lisboa, Portugal) and visualized using a Bio-Rad Gel Doc XR imaging system (Bio-Rad, Hercules, CA, USA). Amplicons were purified and sequenced on an ABI 3730xl DNA Analyzer (96 capillary system) at STABVIDA, Portugal [52].

DNA sequences were quality-checked using Chromas v.2.6.6 (Technelysium, Southport, QLD, Australia). Forward and reverse reads were aligned, assembled into consensus sequences, examined for ambiguous bases, and trimmed at both ends using BioEdit v.7.2.5 [53]. The resulting consensus sequences were deposited in GenBank, with accession numbers listed in Supplementary Table S2. Sequence similarity searches were performed against the NCBI nucleotide database using BLASTN v.2.6 [39] under the MEGABLAST option. Based on preliminary BLAST results, six datasets were constructed to assess the phylogenetic placement of the isolates. These included individual alignments for ITS, LSU, SSU, *RPB2*, and *TEF1α*, as well as a concatenated dataset incorporating all five loci. The resulting multilocus dataset was used to further assess the phylogenetic placement of the isolates, providing molecular support for the preliminary identification and complementing the morphological characterization.

Sequences of each region were individually aligned using the online version of MAFFT v.7 [54], and alignments manually adjusted and concatenated using MEGA software v.11.0.11 [55]. Prior to the phylogenetic analysis, the model of nucleotide substitution was estimated under the Akaike Information Criterion (AIC) using ModelFinder [56] on the W-IQ-TREE v.2.4.0 webserver [57]. The best-fit models for each partition and statistics are provided in Supplementary Table S3. Maximum likelihood (ML) analyses were performed on the same web server, with branch support assessed using 1000 replicates of ultrafast bootstrap analysis [58], the Shimodaira-Hasegawa-like approximate likelihood ratio test (SH-aLRT) [59], and the approximate Bayes test (aBayes) [60].

All alignment matrices and tree files were deposited in Figshare under DOI 10.6084/m9.figshare.30566054. Phylogenetic trees were visualized and edited using FigTree v.1.2.2, MEGA v.11.0.11, and Microsoft PowerPoint 2016.

### 2.5. Genealogical Concordance Phylogenetic Species Recognition (GCPSR)

GCPSR analyses based on the pairwise homoplasy index (PHI; Φw) were performed in SplitsTree4 [61] to assess recombination among phylogenetically closely related taxa and to provide additional support for species delimitation [62]. The analysis included the newly generated strains of *Periconia callaina* examined in this study together with the strains of the most closely related species [63]. A concatenated dataset comprising ITS, LSU, SSU, *RPB2*, and *TEF1α* sequences was used for the PHI test and subsequent split network analyses. Relationships among the newly generated strains and their closest relatives were visualized using split networks based on the LogDet transformation and NeighborNet algorithm. PHI values equal to or greater than 0.05 (Φw ≥ 0.05) indicated no significant evidence of recombination within the dataset, supporting genetic differentiation among the analysed taxa. In contrast, PHI values below 0.05 (Φw < 0.05) indicated significant recombination, suggesting genetic exchange among the analysed strains.

## 3. Results

### 3.1. Culturable Fungi Diversity

**Figure 2:**
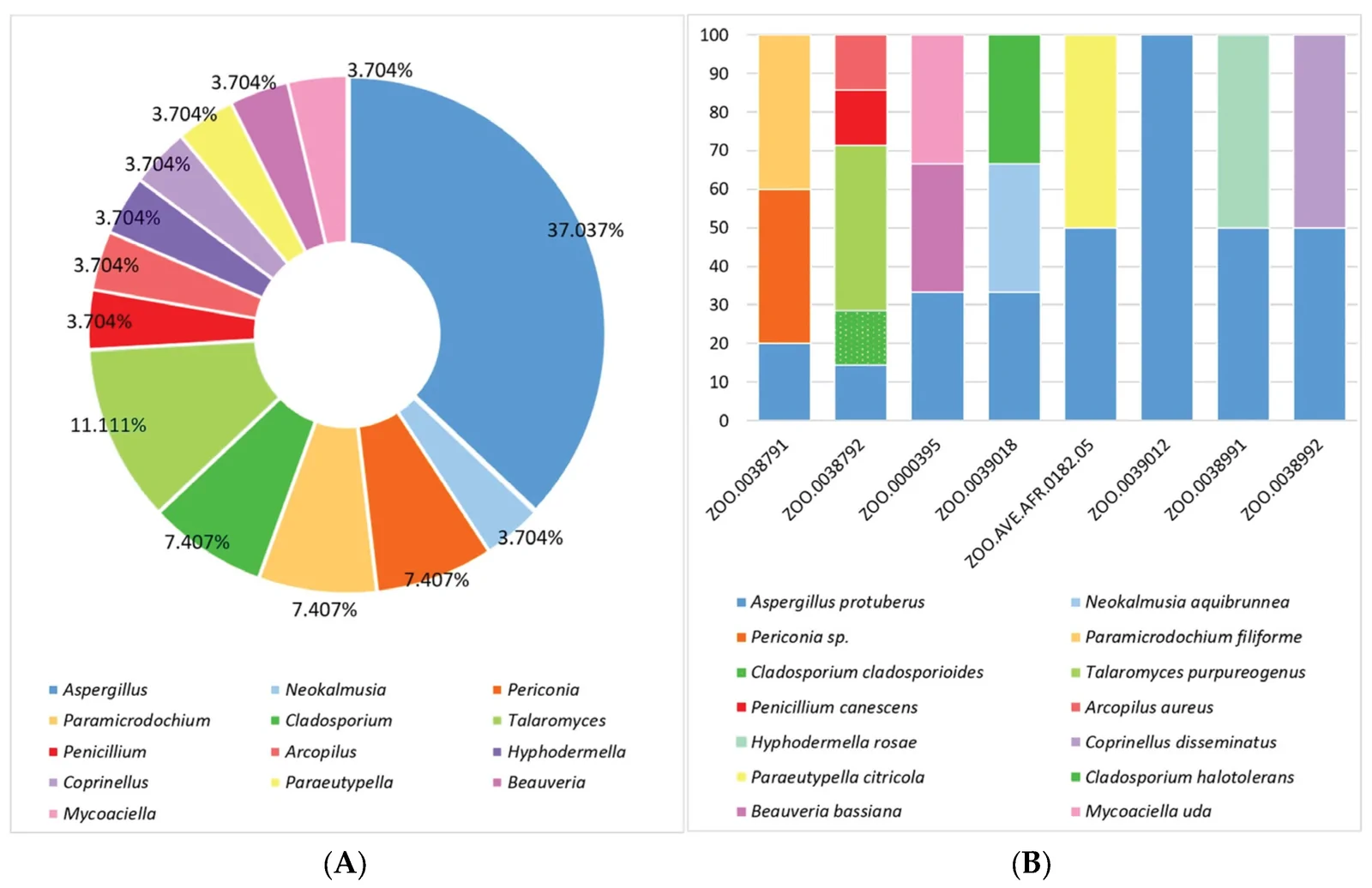
Taxa identified through culture-dependent methods. (**A**) Relative abundance of genera across all samples; (**B**) species identification and relative abundance per sample.

A total of 27 fungal isolates were obtained across all eight samples. Seven isolates were recovered from ZOO.0038792, five from ZOO.0038791, three from ZOO.0000395, ZOO.0039018, and ZOO.0039012, and two each from ZOO.AVE.AFR.0182.05, ZOO.0038991, and ZOO.0038992. Molecular analysis indicated that, of these 27 isolates, 25 belonged to 13 different previously described species. The majority (approximately 37%) were identified as *Aspergillus protuberus* Munt.-Cvetk. (Figure 2), with no other *Aspergillus* species detected. *Talaromyces* was the second most represented genus (11.11%), with *T. purpureogenus* (Stoll) Samson, Yilmaz, Houbraken, Spierenburg, Seifert, Peterson, Varga & Frisvad. The genera *Periconia*, *Paramicrodochium* and *Cladosporium* each accounted for 7.41% of the isolates, with *Paramicrodochium* being represented by the recently described *P. filiforme* D.S. Paiva [52], *Cladosporium* by *C. cladosporioides* (Fresen.) G.A. de Vries and *C. halotolerans* Zalar, de Hoog & Gunde-Cim., the only genus with more than one species identified, and finally *Periconia* with one unidentifiable species. The remaining taxa were *Arcopilus aureus* (Chivers) X.Wei Wang & Samson, *Beauveria bassiana* (Bals.-Criv.) Vuill., *Coprinellus disseminatus* (Pers.) J.E. Lange, *Hyphodermella rosae* (Bres.) Nakasone, *Mycoaciella uda* (Fr.) C.L. Zhao, *Neokalmusia aquibrunnea* J. Yang, Jian K. Liu & K.D. Hyde, *Paraeutypella citricola* (Speg.) L.S. Dissan., Wijayaw., J.C. Kang & K.D. Hyde, and *Penicillium canescens* Sopp, where each represented 3.70% of the total isolates.

**Figure 3:**
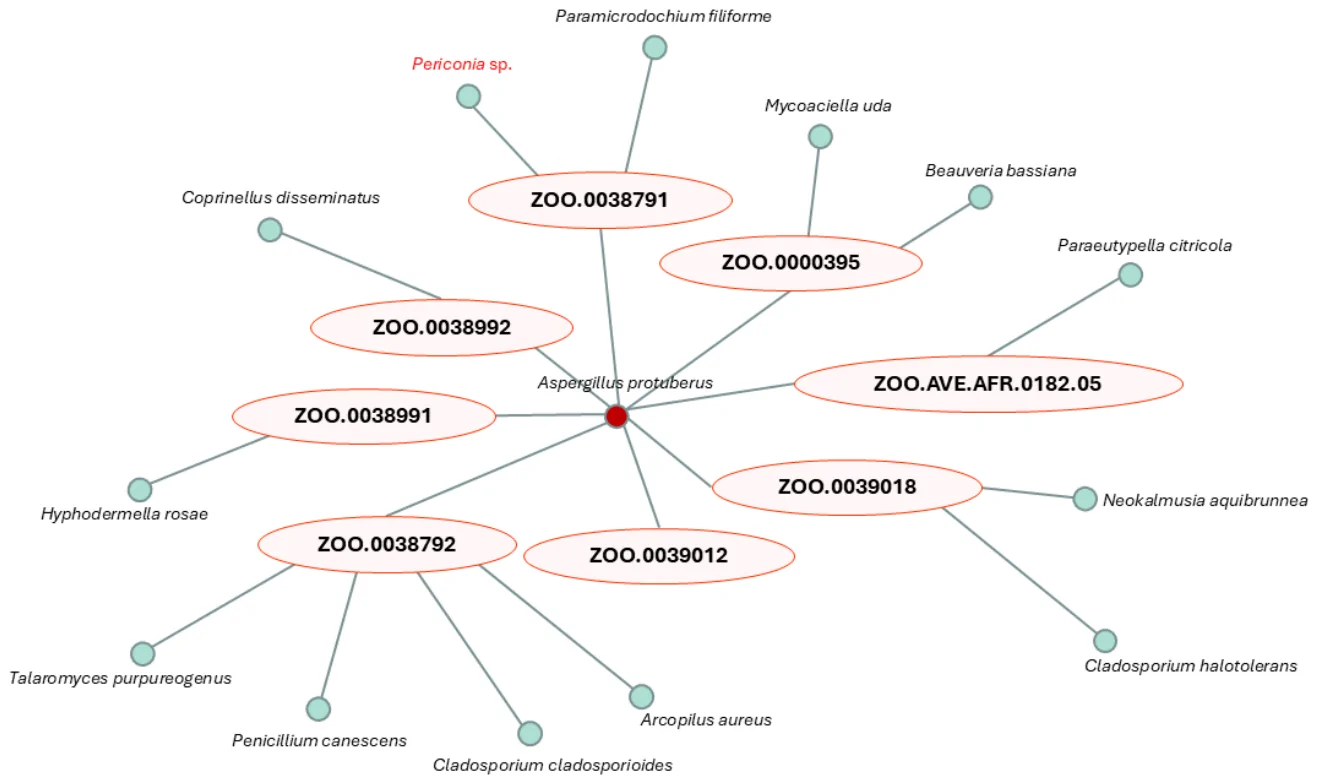
Network analysis showing the co-occurrence patterns of the cultivable species among samples. ZOO.0038791, ZOO.0038792: *Turdoides jardineii tamalakanei*; ZOO.0000395: *Nectarinia bocagii*; ZOO.0039018, ZOO.0039012, ZOO.AVE.AFR.0182.05: *Chalcomitra senegalensis saturatior*; and ZOO.0038991, ZOO.0038992: *Melaenornis pammelaina pammelaina*.

*Aspergillus protuberus* was the only species present across all samples, whereas the remaining fourteen species were exclusively associated with a single sample. Specimen ZOO.0038792 accommodated the highest diversity, with four other species in addition to *A. protuberus* (*Arcopilus aureus*, *Cladosporium cladosporioides*, *Penicillium canescens* and *Talaromyces purpureogenus*). Sample ZOO.0038791 displayed two additional species, *Periconia* sp. and *Paramicrodochium filiforme*, as did ZOO.0000395 (*Beauveria bassiana* and *Mycoaciella uda*) and ZOO.0039018 (*Neokalmusia aquibrunnea* and *Cladosporium halotolerans*). ZOO.AVE.AFR.0182.05, ZOO.0038991, and ZOO.0038992 were only found to contain one additional species each, namely *Paraeutypella citricola*, *Hyphodermella rosae*, and *Coprinellus disseminatus*, respectively. Finally, ZOO.0039012 yielded only *Aspergillus protuberus* (Figure 3). Overall, the isolated fungi were largely sample-specific, with limited overlap between specimens, suggesting a high degree of spatial heterogeneity and potentially localized sources or dispersal processes influencing their distribution.

### 3.2. Phylogenetic Analysis

Initial comparisons with sequences available in the NCBI database were conducted to determine the generic placement of the studied isolates. The ITS, LSU, SSU, and *TEF1α* loci consistently showed high sequence similarity (97–99%) with several *Periconia* species, including *P. macrospinosa* Lefebvre & Aar.G. Johnson (isolates S1c, 107, A2S4-D47, NWU47, among others), *P. epilithographicola* Coronado-Ruiz, Avendaño, Escudero-Leyva, Conejo-Barboza, P. Chaverri & Chavarría (MFLUCC 21-0153), *P. lateralis* Ellis & Everh. (CBS 292.36), *P. genistae* Lunghini (CBS 322.79), *P. algeriana* Lunghini (CBS 321.72), *P. byssoides* Pers. (isolates C457, 202007001, w282, C445B, among others), *P. pseudobyssoides* Markovsk. & A. Kačergius (KUNCC 23-13932, KUMCC 20-0263), *P. wurfbainiae* C.F. Liao, Doilom & K.D. Hyde (ZHKUCC 23-0999, ZHKUCC 23-1000), and *P. circinata* (L. Mangin) Sacc. & D. Sacc. (CBS 263.37), as well as several unidentified *Periconia* species and other *Pleosporales* isolates. In contrast, the *RPB2* locus exhibited only distant matches (≤88% similarity), primarily with *P. byssoides* (MFLUCC 18-1555, MFLUCC 18-1553, among others) and *P. cynodontis* Z.Hua Lu, P.W. Su & Maharachch. (isolate Lu4). These results strongly suggested an affiliation with *Periconia* genus; however, definitive taxonomic placement remained unresolved.

**Figure 4:**
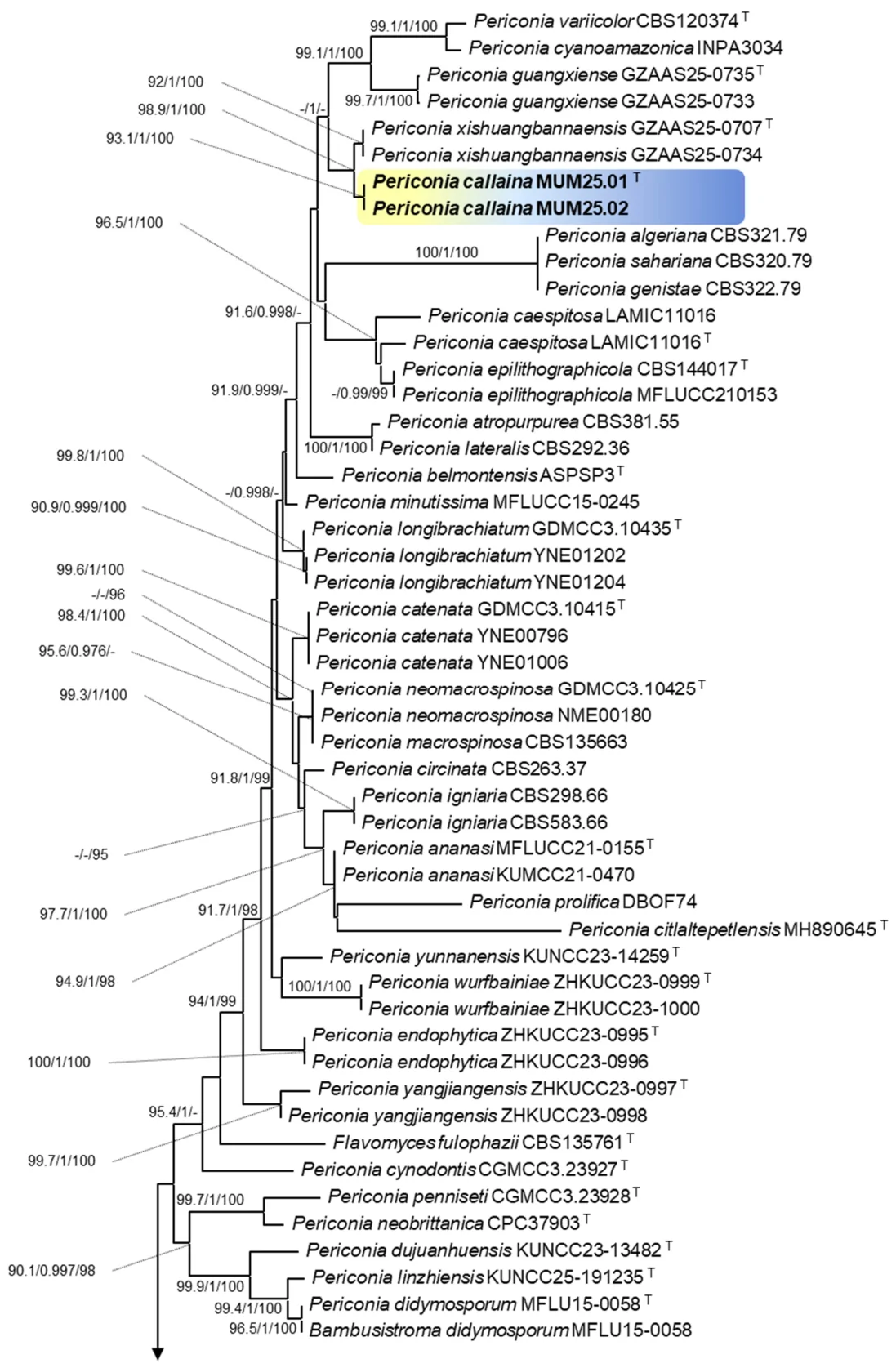

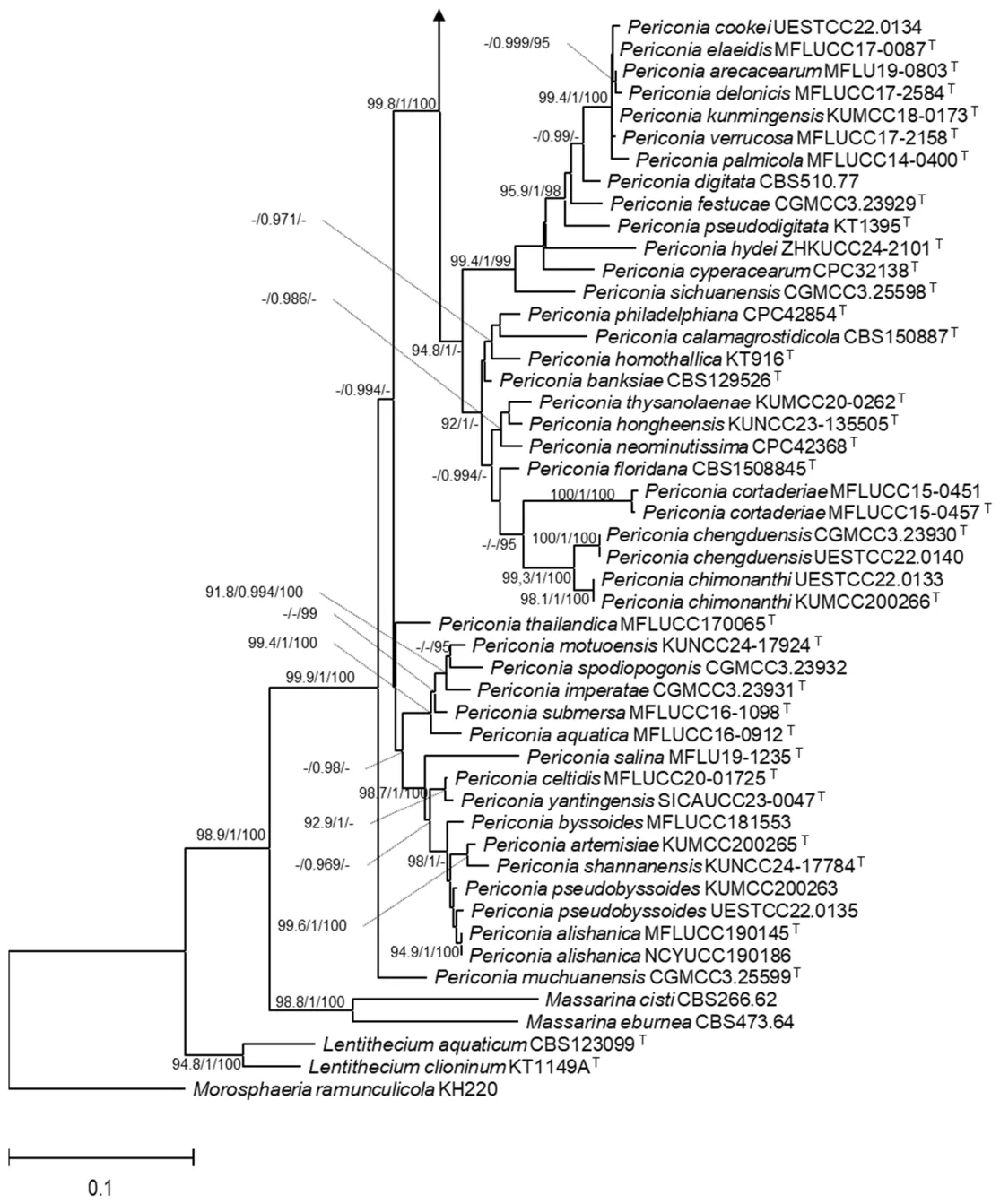
Maximum likelihood (ML) tree based on concatenated sequence data from ITS, LSU, SSU, *RPB2* and *TEF1α* showing the placement of the new species, *Periconia callaina*, described in this study. The scale bar indicates the number of substitutions per site. Nodes are labelled with SH-aLRT/aBayes/ultrafast bootstrap support values ≥ 90%/0.95/95%, with type strains designated by superscript T. The novel species is highlighted in bold font. The tree is rooted with *Morosphaeria ramunculicola* (KH220).

Phylogenetic relationships were inferred using a concatenated dataset of five loci, comprising 3452 aligned characters (471 for ITS, 747 for LSU, 1022 for SSU, 514 for *RPB2*, and 698 for *TEF1α*, including alignment gaps). The optimal nucleotide substitution models for each of the loci are listed in Supplementary Table S3. The resulting tree (Figure 4) was consistent with existing knowledge, with overall topology aligning with recent taxonomic studies regarding this genus [45,46,47,48,49,50,51]. The phylogenetic analysis demonstrated that the studied isolates resolved in a distinct, strongly supported monophyletic branch (93.1% SH-aLRT, 1 aBayes, 100% bootstrap support), sister to *P. xishuangbannaensis* X.G. Tian & D.F. Bao. Both species occupied a strongly supported cluster (98.9% SH-aLRT, 1 aBayes, 100% bootstrap support) closely related to the cluster encompassing *P. variicolor* S.A. Cantrell, Hanlin & E. Silva, *P. cyanoamazonica* I.C. Bandeira, and *P. guangxiense* X.G. Tian & D.F. Bao. These taxa collectively formed an independent clade (87.9% SH-aLRT, 1 aBayes, 90% bootstrap support), distinct from other *Periconia* species. Additionally, independent phylogenetic analyses of ITS, LSU, SSU, *RPB2*, and *TEF1α* loci supported these findings, further confirming the novelty of the studied isolates (Supplementary Figures S1–S5). Based on this evidence, we propose the establishment of *Periconia callaina* sp. nov. to accommodate this newly identified species.

**Figure 5:**
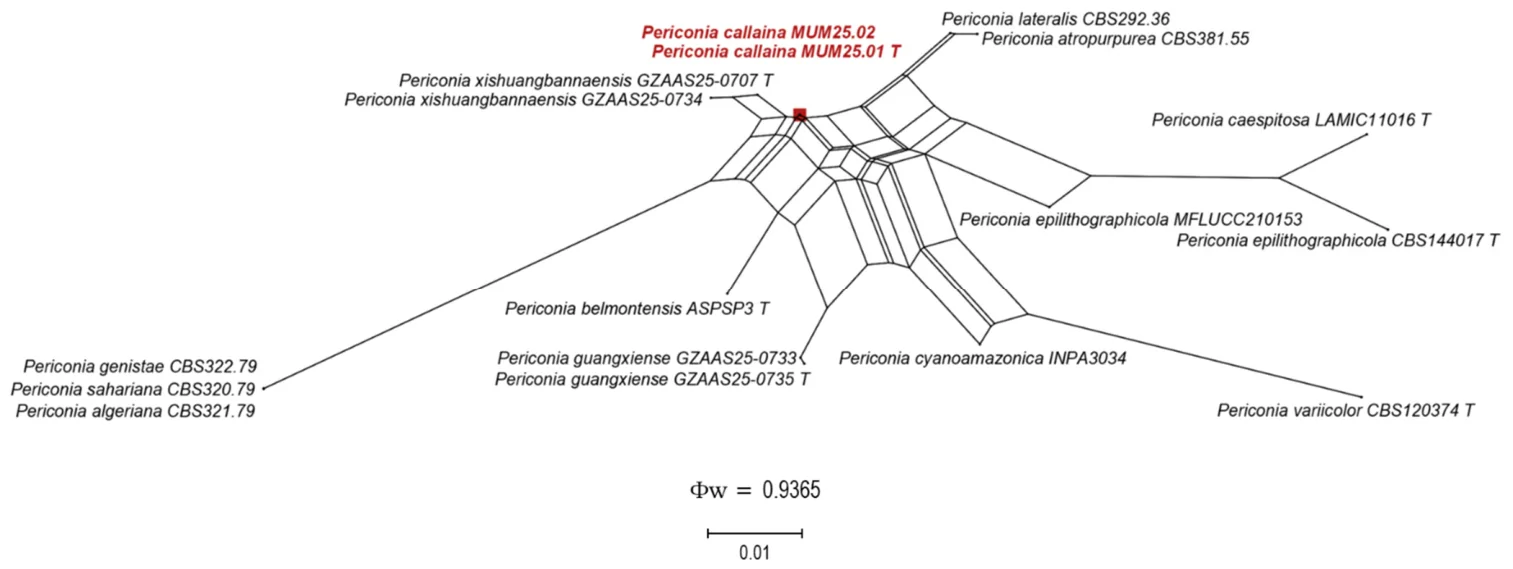
Split network from the PHI test based on combined ITS, LSU, SSU, *RPB2*, and *TEF1α* sequences of *Periconia callaina* (in red) and related taxa, inferred using LogDet transformation and the NeighborNet algorithm. Φw > 0.05 indicates no significant recombination.

The PHI test based on the concatenated ITS, LSU, SSU, *RPB2*, and *TEF1α* dataset showed no significant evidence of recombination between the *Periconia callaina* isolates and representative strains of phylogenetically closely related species (Φw = 0.9365), further supporting their genetic differentiation from the closely related taxa and supporting their recognition as a distinct species within *Periconia* (Figure 5).

### 3.3. Taxonomy

**Figure 6:**
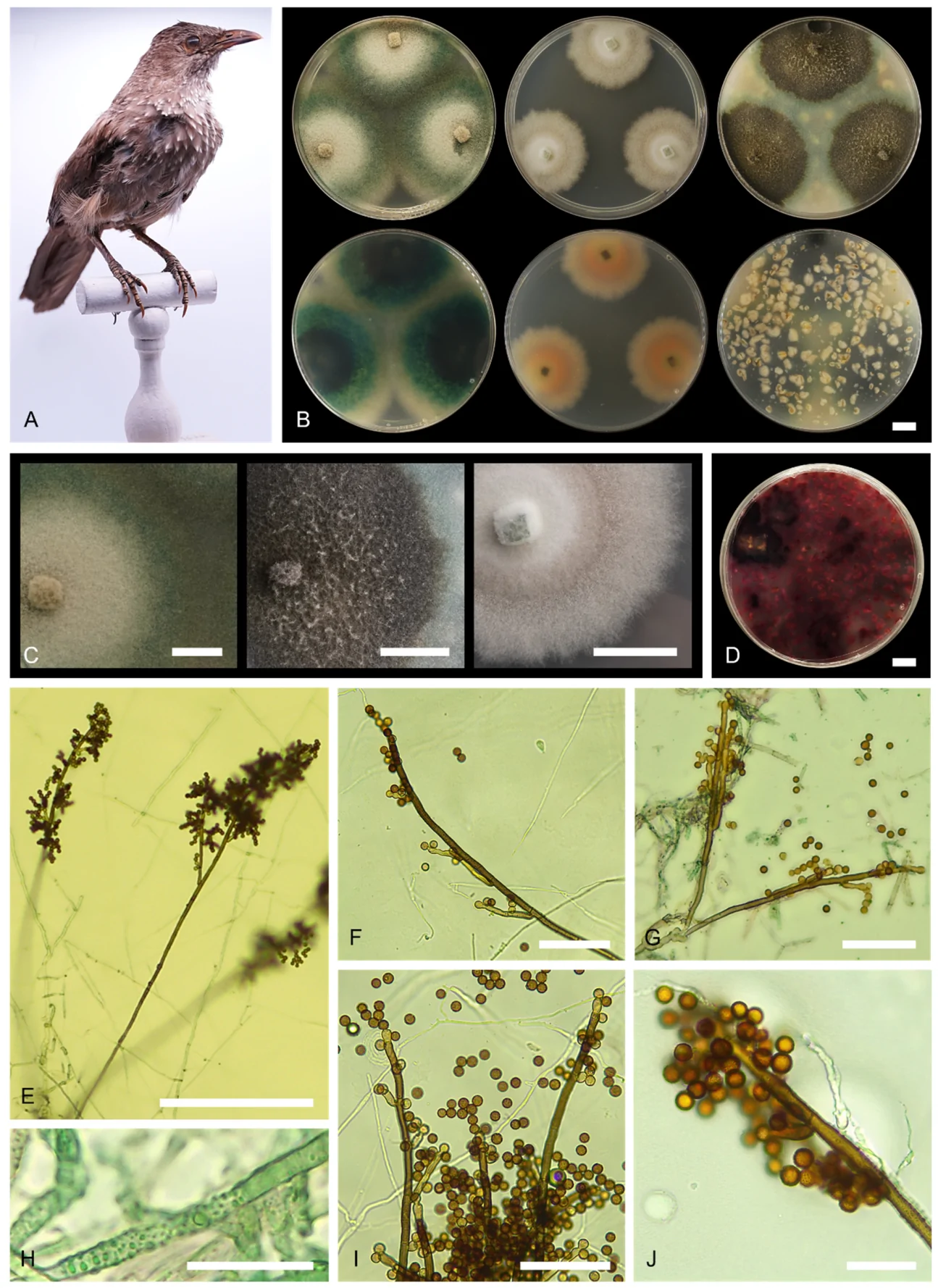
*Periconia callaina* (MUM 25.01, ex-type). (**A**). *Turdoides jardineii tamalakanei*, specimen no. ZOO.0038791, from which this fungal species was isolated. (**B**). Colony morphology on PDA, MEA, and OA, respectively. (**C**). Colony details on PDA, OA, and MEA, respectively. (**D**). Red pigment in older colony on OA. (**E**–**G**,**I**,**J**). Conidiophores and conidia. (**H**). Detail of verrucose hyphae. Scale bars: (**B**–**D**) = 1 cm; (**E**) = 200 µm; (**F**,**G**,**I**) = 50 µm; (**H**,**J**) = 20 µm.

***Periconia callaina***** L. Fernandes & D.S. Paiva, sp. nov.,** Figure 6**.**

**MycoBank MB858472**

**Etymology:** “*Callaina*” from Latin, meaning greenish-blue or turquoise, referring to the characteristic coloration of the colonies in PDA.

**Classification:** division *Ascomycota*, class *Dothideomycetes*, subclass *Pleosporomycetidae*, order *Pleosporales*, family *Periconiaceae*, genus *Periconia*.

***Typus*****:** Portugal, Coimbra, isolated from a taxidermized bird (*Turdoides jardineii tamalakanei*, specimen no. ZOO.0038791) at the Science Museum of the University of Coimbra, 2 April 2024, L. Fernandes (holotype MUM-H 25.01, dried specimen); ex-type culture MUM 25.01.

**Description:** Spore masses on SNA after 4 wk scattered, appearing as black spots on colonies. *Hyphae* hyaline when young turning moderate yellowish green (ISCC-NBS No. 136) when ageing, septate, verrucose, 2–4 µm wide (mean = 3 µm, *n* = 30). *Conidiophores* arising from substrate mycelium, dark olive brown (ISCC-NBS No. 96), 295–645 µm long (mean = 430, *n* = 30), 3–5 µm wide (mean = 3.7 µm, *n* = 30), macronematous, mononematous, straight or slightly flexuous, branched, solitary or gregarious, septate, verrucose, thick-walled. *Conidiogenous cells* polyblastic, light olive brown (ISCC-NBS No. 94), terminal, integrated, oblong, with rounded apex, verrucose. *Conidia* globose, 4.5–7 µm (mean = 5.8 µm, *n* = 30), dark olive brown (ISCC-NBS No. 96), aseptate, verrucose, thick-walled, solitary or catenate. Sexual morph unknown.

**Culture diameter, 3 weeks (in mm):** PDA 60–65; MEA 35–40; OA 55–60.

**Culture characteristics:** PDA 25 °C, 3 wk: Colonies circular, dense, lightly raised at centre, with concentric zonation in the middle region, margins filamentous, slightly fluffy; mycelium white (ISCC-NBS No. 263) at centre, transitioning into strong bluish green (ISCC-NBS No. 160) to moderate yellow green (ISCC-NBS No. 120) towards the margin, texture cottony, smooth; exudate absent; soluble pigment absent; reverse colour very dark bluish green (ISCC-NBS No. 166) at centre fading into strong bluish green (ISCC-NBS No. 160) with a white margin (ISCC-NBS No. 263). MEA 25 °C, 3 wk: Colonies circular, dense, flat; margins narrow, filamentous; mycelium white (ISCC-NBS No. 263) at centre, pale greenish yellow (ISCC-NBS No. 104) from the middle region to the margin; texture cottony, smooth; with slight soluble pigment present, vivid red (ISCC-NBS No. 11); exudate absent; reverse colour pale yellow (ISCC-NBS No. 89). OA 25 °C, 3 wk: Colonies circular, moderately deep, flat, margins filamentous, slightly fluffy; mycelium sparce, white (ISCC-NBS No. 263); texture cottony to floccose; sporulation dense, conidia *en masse* dark greyish olive (ISCC-NBS No. 111); exudate absent; soluble pigment absent; reverse colour dark greyish olive (ISCC-NBS No. 111).

**Additional specimens examined:** Portugal, Coimbra, isolated from a taxidermized bird (*Turdoides jardineii tamalakanei*, specimen no. ZOO.0038791) at the Science Museum of the University of Coimbra, 2 April 2024, L. Fernandes, MUM 25.02.

**DNA barcodes:** MUM 25.01 ITS: PV341358; LSU: PV387312; SSU: PV387571; *RPB2*: PV423526; *TEF1α*: PV423528. MUM 25.02 ITS: PV341359; LSU: PV387313; SSU: PV387572; *RPB2*: PV423527; *TEF1α*: PV423529.

**Notes:** *Periconia callaina* exhibits the characteristic morphology of *Periconia* species, forming macronematous, mononematous conidiophores with a spherical conidial head, polyblastic conidiogenous cells, and catenate, globose, brown, aseptate, verruculose conidia. Phylogenetic analyses based on concatenated ITS, LSU, SSU, RPB2, and TEF1α sequences strongly support its placement within *Periconia*, positioning *P. callaina* on an independent branch, sister to *P. xishuangbannaensis*, and closely related to the clade containing *P. variicolor*, *P. guangxiense* and *P. cyanoamazonica*, the latter two also being recent additions to the genus (Figure 4). Morphologically, *P. callaina* is distinguished by its characteristic blue-green coloration, which is also evident at the micromorphological level, particularly in its hyphae, that appear to contain similarly coloured vesicle-like structures. A comparable pigmentation has been reported in *P. cyanoamazonica* [64], although the overall micromorphology differs. Like *P. caespitosa* Cantillo, Gusmão & Madrid, *P. variicolor*, and *P. guangxiense* it produces a deep red pigment, but in *P. callaina*, this feature is observed only in older colonies [49,65,66]. Additionally, its conidiophores exceed 600 µm in length, whereas *P. xishuangbannaensis* reach an average of 243 µm, *P. epilithographicola* and *P. variicolor* approximately 270 µm, and *P. caespitosa* up to 500 µm [46,49,65,66,67]. In contrast, *P. callaina* tends to produce slightly smaller conidia, measuring up to 7 µm, while the conidia of these related species range from 9 to 10 µm, with the exception of *P. guangxiense* and *P. xishuangbannaensis*, which show partially overlapping size ranges, 6–7.5 μm and 4.5–7 μm respectively. Another distinguishing feature of *P. callaina* is its branched conidiophores, a trait it shares with *P. epilithographicola* [46], *P. guangxiense* and *P. xishuangbannaensis* [49]. Notably, *P. callaina* was isolated from a taxidermized animal, marking the first known occurrence of this genus in such a context. *Periconia* species are widely distributed, predominantly in terrestrial habitats, with most being saprophytes or endophytes, while a few are plant pathogens. The species most similar in terms of environmental context is *P. epilithographicola*, which was isolated from a university repository of drawings and lithographs, an environment significantly affected by fungal biodeterioration [67].

## 4. Discussion

### 4.1. Culturable Fungi Diversity

In the present study, taxidermized birds from the MCUC were found to harbour fungi previously reported from similar cultural heritage assets. However, assessing fungal contamination in historical taxidermized specimens presents several challenges, particularly in terms of sampling representativeness. The non-invasive approach adopted in this study was necessary to minimise disturbance to the specimens, but sampling was necessarily restricted to accessible areas showing visible fungal growth. Given the heterogeneous composition of taxidermized specimens and the potentially uneven distribution of fungal propagules across their surfaces, the isolates recovered may not represent the entire fungal community associated with each specimen. In addition, visible fungal growth may account for only part of the fungal propagules present, while fungi occurring in less accessible or apparently unaffected areas may remain undetected. Although multiple fungal taxa were recovered from individual specimens, the limited number of specimens examined for each bird species, together with the influence of shared museum environmental conditions, prevents any conclusions regarding species-specific fungal associations, which remain an interesting avenue for future research.

Furthermore, the presence of fungi on a specimen does not necessarily imply established colonization or active biodeterioration. Museum specimens are continuously exposed to airborne fungal propagules, making it difficult to distinguish transient environmental contamination from fungi that have become established on the specimen. The culture-dependent approach used in this study may also favour organisms able to grow under the selected culture conditions, potentially overlooking fungi with different nutritional or physiological requirements. Therefore, the fungal diversity recovered in this study should be regarded as the cultivable fraction associated with the visibly affected areas sampled, rather than as a complete representation of the mycobiota of the preserved specimens. Despite these limitations, the recovery of fungi previously associated with cultural heritage materials highlights the value of targeted, non-invasive sampling as a first approach for assessing fungal occurrence in historical museum collections.

*Aspergillus protuberus* was the only species present in all samples, and this may suggest that it was the main fungus responsible for the outbreak observed in the collection. The predominance of the *Aspergillus* genus in this museum’s environment had already been observed in our previous work concerning a different collection, in a different room [24]. However, no overlap of species was observed between both works, and while fewer species were observed in this current assessment, a wider range of genera (13) was detected when compared to our work in the Anthropology archive, where only 6 genera were identified.

Already associated with organic acid production, calcium carbonate dissolution and mineralization, and the production of ligninolytic, fibrinolytic, keratinolytic, estereolytic, lipolytic, proteolytic and cellulolytic activity [68,69], the *Aspergillus* genus is of particular concern in the context of biodeterioration. *Aspergillus protuberus* has been previously found to be contaminating cultural heritage artifacts such as silk textiles [70], books [71], limestone monuments [72], and paintings [73]. It has well-reported cellulolytic and proteolytic abilities [68] and produces extracellular pigments [74].

Also commonly reported in museum environments and cultural heritage settings, the *Talaromyces*, *Cladosporium* and *Penicillium* genera were also identified in this study, with the isolated species, *T. purporeogenus*, *C. cladosporioides*, *C. halotolerans* and *P. canescens*, having previous associations with cultural heritage [8,15,17,72,75,76,77,78,79,80,81,82,83,84,85,86,87,88]. These species have also been described as possessing deteriorative potential including, cellulolytic, estereolytic, proteolytic, and ligninolytic activity, alongside organic acid and pigment production [68,89,90,91,92]. Of particular concern is *C. cladosporioides* known keratinolytic ability [68], considering the context of this work and the taxidermized birds sampled. Keratinases, including those produced by *C. cladosporioides*, have the ability to degrade feathers [93,94]. As such, the presence of this fungus in this collection represents a significant potential risk to its integrity.

The discovery of *Beauveria bassiana* is also of particular concern. Although it is primarily known as an entomopathogen, this species has been previously isolated from wall paintings in China [95], and has known ligninolytic, fibrinolytic, proteolytic, cellulolytic, lipolytic, chitinolytic, cellulolytic and, concerningly, keratinolytic capabilities [68,96]. While it has also been used as conservation agent for metal artifacts due to its ability to transform corrosion products into metal-oxalate complexes [97,98,99], the above-described abilities pose a severe threat not only to the integrity of the collection from which this species was isolated, but also to others across the museum. Its entomogenous nature can also provide an insight into the potential processes through which it entered and/or propagated inside the museum, as it also struggles with occasional insect outbreaks.

Of the identified fungal isolates, and to the best of our knowledge, four ascomycetes were here associated with museum environments for the first time, namely *Arcopilus aureus*, *Neokalmusia aquibrunnea*, *Paraeutypella citricola* and *Paramicrodochium filiforme*. *Arcopilus aureus* is an endophytic fungus and a known pigment producer [100]. *Neokalmusia aquibrunnea* is a saprobic fungus described by Yang and colleagues [101] from decaying, submerged bamboo culms, later isolated from decaying branches of chinese windmill palm, *Trachycarpus fortunei* [102]. This present work marks only the third report of detection of this species. *Paraeutypella citricola* has been previously isolated from dead branches and woody litter [103,104,105]. A recent addition to the *Sordariomycetes incertae sedis* class, *Paramicrodochium filiforme* was first described by Paiva and colleagues from a deteriorated limestone monument [52]. To the best of our knowledge, this is only the second time this species has been isolated.

Three basidiomycetes, *Coprinellus disseminatus*, *Hyphodermella rosae*, and *Mycoaciella uda* were found in this study. Typically associated with the degradation of wooden artifacts [8,106], basidiomycetes too are common contaminants of cultural heritage. In fact, of the three *Basidiomycota* species identified, only *M. uda* has no previous reports of contamination of cultural heritage. Both *C. disseminatus* and *H. rosae* have been associated with the deterioration of paper and parchment documents [18,107,108], with *C. disseminatus* also being isolated from wooden iconostasis in a cave church in Serbia [109]. Both species have been reported to be ligninolytic [108], with *C. disseminatus* also being a known cellulase, proteinase, and lipase producer [107].

The distribution of the recovered fungi among the sampled specimens further highlights the heterogeneity of the fungal community associated with the taxidermized birds. Specimen ZOO.0038792 harboured the highest fungal diversity, with *Aspergillus protuberus*, *Arcopilus aureus*, *Cladosporium cladosporioides*, *Penicillium canescens* and *Talaromyces purpureogenus* recovered, whereas other specimens harboured fewer taxa, with ZOO.0039012 yielding only *A. protuberus*. The remaining specimens showed distinct combinations of fungal taxa, with limited overlap between samples. This largely specimen-specific distribution suggests considerable spatial heterogeneity in fungal occurrence and may reflect localized environmental conditions, deposition, or dispersal processes within the collection. While fungal growth was mainly observed in the feathers, the multi-substrate nature of the sampled specimens, alongside the potential for the recovered isolates to be present merely as propagules limits the ability to establish clear relationships between individual fungal taxa and the substrate from which they were recovered. Nevertheless, the presence of taxa with known biodeteriorative potential, particularly those capable of keratinolytic activity, on individual specimens highlights the importance of considering specimen-specific fungal occurrence when assessing potential risks to taxidermized collections.

Taking all this into consideration, the presence of these fungi constitutes a severe threat not only to this collection, but also to all other collections of the MCUC. Also important to note is the potential health risk caused by the presence of these contaminants, with genera such *Aspergillus* and *Penicillium*, here identified, being known for their ability to cause respiratory tract infections and other clinical manifestations [23,110,111].

Additionally, this study constitutes, to the best of our knowledge, the first record of *A. aureus*, *M. uda*, *N. aquibrunnea*, *P. citricola*, and *P. filiforme* being isolated from museum artifacts, providing further insight into their adaptability and potential substrates. Finally, several identified species have never been evaluated for their biodeteriorative potential, with further studies being essential for a better understanding of their impact on cultural heritage.

### 4.2. Description of a New Periconia Species

In the present study, the taxonomic status of two unidentified *Periconia* isolates obtained from a taxidermized bird specimen in the MCUC collection was investigated using an integrative approach. The combined morphological, phylogenetic, and GCPSR analyses supported their recognition as a distinct species, which is here introduced as *Periconia callaina*.

The phylogenetic analysis placed the two isolates in a distinct and well-supported lineage within *Periconia*, closely related to *P. xishuangbannaensis*. The strong support for the nodes defining the *P. callaina* lineage, together with the morphological differences observed between *P. callaina* and its closest relatives, provides robust evidence for its recognition as a separate species. Species delimitation was further supported by the GCPSR analysis, which showed no significant evidence of recombination between the newly proposed species and closely related taxa, providing independent support for the inferred species boundaries. Despite this well-supported placement, the broader relationships within *Periconia* should be interpreted with some caution. The genus has a complex taxonomic history and has repeatedly been shown to be polyphyletic, while relationships among several species remain unresolved or weakly supported [47]. Recent studies have also emphasized that improved taxon sampling and the inclusion of additional protein-coding loci are needed to obtain a clearer understanding of species relationships and phylogenetic boundaries within the genus [49].

First introduced in 1791 by H. J. Tode, the genus *Periconia* is a polyphyletic one, and is classified as a member of the *Periconiaceae* and separated from *Massarineae* as a sister taxon, with distinct phylogeny [112]. Typically known for their asexual morphs, *Periconia* species are characterised by macronematous, mononematous conidiophores, and globose to ellipsoidal conidia, while the sexual morph is characterised by immersed to erumpent, scattered or aggregated, subglobose to globose ascomata, hyaline, 8-spored, bitunicate, cylindrical, septate, guttulate, ascospores with an entire sheath [113]. While Index Fungorum (www.indexfungorum.org) notes the existence of 182 currently accepted species of this genus, most of these do not have phylogenetic marker data available and as such were unable to be included in our analysis beyond morphology. However, *P. callaina* presents very distinct morphological features, particularly its characteristic blue-green coloration, a trait only displayed by *P. cyanoamazonica*, a species that our phylogenetic analysis places in a separate clade to that of *P. callaina*. Future revisions may be necessary as molecular data becomes available for these formerly described species or for additional newly identified species. Nevertheless, with the most current information, *P. callaina* stands as a new addition to this ever-growing genus.

Species from this genus have been reported as saprobes and endophytes, as well as both plant and human pathogens [46,47] with reports of *Periconia* species causing mycotic keratitis [114]. Two species of the *Periconia* genus have also been described as cultural heritage contaminants, namely *P. epilithographicola*, which was discovered and described as a contaminant of a 19th century artwork with cellulolytic capabilities [67], and *P. macrospinosa*, found in a biofilm on the interior of a gothic church in Southern Italy [115].

The environmental context from which *P. callaina* was isolated is also of relevance. A taxidermized bird stored in a museum represents an ecological niche new to the genus as a whole, thus providing new insights into the adaptability of *Periconia* and the substrates it can be found in. Furthermore, the biodeteriorative potential of this new species remains a mystery, and a further evaluation of these characteristics is key in understanding its potential role as a cultural heritage contaminant, even though it has already shown itself as a strong pigment producer.

Finally, the *Periconia* genus has been previously reported as a strong producer of bioactive secondary metabolites, with activity spectrums as wide as antimicrobial, anti-human immunodeficiency virus (HIV), and anti-inflammatory, among others [112]. As such, a new addition to this genus represents an opportunity for new, naturally occurring secondary compounds to be explored for their biotechnological potential.

## 5. Conclusions

In the present work, the fungal contamination observed in a collection of taxidermized birds from the Science Museum of the University of Coimbra was studied. This analysis led to the detection of 27 isolates, belonging to 13 different genera and 14 different species. Most of these have been previously associated with cultural heritage contamination and deterioration, with several isolates possessing capabilities that could compromise not only the sampled collection, but most of the museum’s artifacts. Of particular concern was the identification of *C. cladosporioides* and *B. bassiana*, for their keratinolytic potential, given the nature of the sampled pieces.

Among the 27 isolates, two were unable to be identified at the species level but were shown to belong to the *Periconia* genus. Further phylogenetic analysis demonstrated that these isolates belonged to a new species, *P. callaina*, here described. This work provides valuable molecular and morphological data that enhance our understanding of fungi in this ever-growing genus. Additional studies of the physiological and biodeteriorative potential of this new taxon are needed to fully understand its role as a contaminant of cultural heritage. Understanding the communities that inhabit and are involved in the deterioration of cultural heritage is key to the development and implementation of safeguarding measures that ensure the preservation of our cultural heritage for future generations.

## Data Availability

All relevant data supporting the findings of this study are included in the article and its Supplementary Information File. The nucleotide sequences have been deposited in the GenBank database under the following accession numbers: PV341358-PV341359, PV484568-PV484589 (ITS); PV387312-PV387313, PV484675-PV484686 (LSU); PV387571-PV387572, PV484690-PV484694 (SSU); PX434372-PX434378; PX438799-PX438800 (*BTUB*); PV423528-PV423529, PX434388 (*TEF1α*); PX434379-PX434386 (*CAM*); and PV423526-PV423527, PX434387 (*RPB2*). The isolates studied in this work are preserved in the Micoteca da Universidade do Minho (MUM), Braga, Portugal, under the references MUM 25.01 and MUM 25.02. Additionally, alignment matrices and phylogenetic tree files are accessible on Figshare with the DOI identifier: 10.6084/m9.figshare.30566054.

## Supplementary Materials

The following supporting information can be downloaded at: https://www.mdpi.com/article/10.3390/microorganisms14091894/s1, Figure S1: Maximum likelihood (ML) tree based on sequence data from ITS showing the placement of the new species, Periconia callaina, described in this study. The scale bar indicates the number of substitutions per site. Nodes are labelled with SH-aLRT/aBayes/ultrafast bootstrap support values ≥ 70%/0.70/70%, with type strains designated by superscript T. The novel species is highlighted in bold font. The tree is rooted with Lentithecium; Figure S2: Maximum likelihood (ML) tree based on sequence data from LSU showing the placement of the new species, *Periconia* *callaina*, described in this study. The scale bar indicates the number of substitutions per site. Nodes are labelled with SH-aLRT/aBayes/ultrafast bootstrap support values ≥ 70%/0.70/70%, with type strains designated by superscript T. The novel species is highlighted in bold font. The tree is rooted with *Morosphaeria ramunculicola *(KH220); Figure S3: Maximum likelihood (ML) tree based on sequence data from SSU showing the placement of the new species, *Periconia callaina*, described in this study. The scale bar indicates the number of substitutions per site. Nodes are labelled with SH-aLRT/aBayes/ultrafast bootstrap support values ≥ 70%/0.70/70%, with type strains designated by superscript T. The novel species is highlighted in bold font. The tree is rooted with *Morosphaeria ramunculicola* (KH220); Figure S4: Maximum likelihood (ML) tree based on sequence data from RPB2 showing the placement of the new species, *Periconia callaina*, described in this study. The scale bar indicates the number of substitutions per site. Nodes are labelled with SH-aLRT/aBayes/ultrafast bootstrap support values ≥ 70%/0.70/70%, with type strains designated by superscript T. The novel species is highlighted in bold font. The tree is rooted with *Massarina cisti* (CBS266.62); Figure S5: Maximum likelihood (ML) tree based on sequence data from TEF1α showing the placement of the new species, *Periconia callaina*, described in this study. The scale bar indicates the number of substitutions per site. Nodes are labelled with SH-aLRT/aBayes/ultrafast bootstrap support values ≥ 70%/0.70/70%, with type strains designated by superscript T. The novel species is highlighted in bold font. The tree is rooted with *Morosphaeria ramunculicola* (KH220); Table S1: Identification of all isolates retrieved in this study; Table S2: Strain collection references and GenBank accession numbers for the novel species outlined in this study, along with additional strains included in phylogenetic analyses, with *Massarina cisti* as the outgroup; Table S3: The best–fit substitution models for each partition determined by ModelFinder based on the Akaike Information Criterion.
