# Supplementary material for: Spores Among Feathers: Evaluating Fungal Diversity in Taxidermized Birds and the Discovery of a New Species
Source: Microorganisms. 2026 Aug 26;14(9):1894. doi: 10.3390/microorganisms14091894 (PMC13609718; doi:10.3390/microorganisms14091894)
Supplement: Supplementary file 1 [file microorganisms-14-01894-s001.zip › Supplementary materials - Figures S1-S5.pdf]

Spores Among Feathers: Evaluating Fungal Diversity in Taxidermized Birds and the Discovery of a New Species

Luís Fernandes [1,†], Diana Paiva [1,*,†], Emília Pereira [1], Célia Cabral [1,2,3,4], Nuno Mesquita [1] and António Portugal [1,5,6]

Supplementary Materials

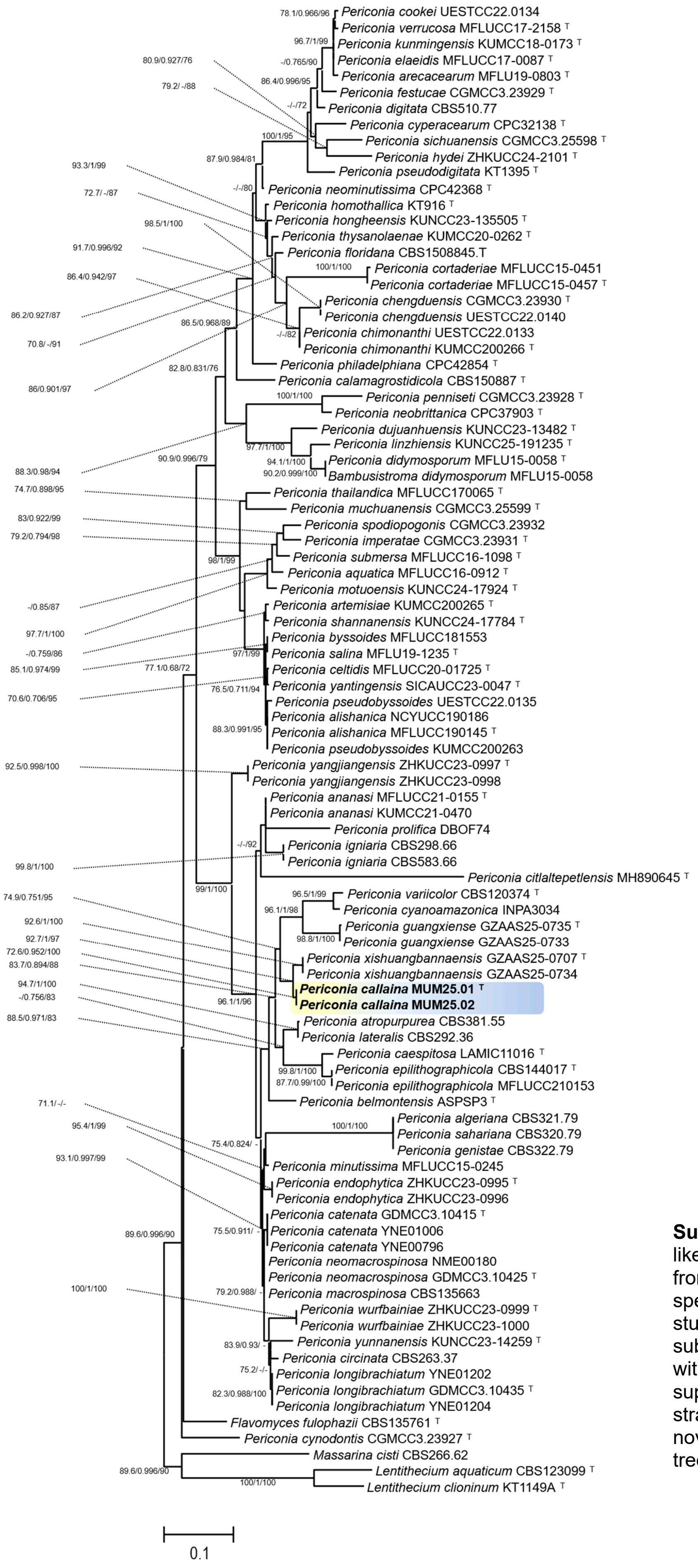

Supplementary Figure S1. Maximum likelihood (ML) tree based on sequence data from ITS showing the placement of the new species, *Periconia callaina*, described in this study. The scale bar indicates the number of substitutions per site. Nodes are labelled with SH-aLRT / aBayes / ultrafast bootstrap support values ≥ 70 % / 0.70 / 70 %, with type strains designated by superscript T. The novel species is highlighted in bold font. The tree is rooted with *Lentithecium*.

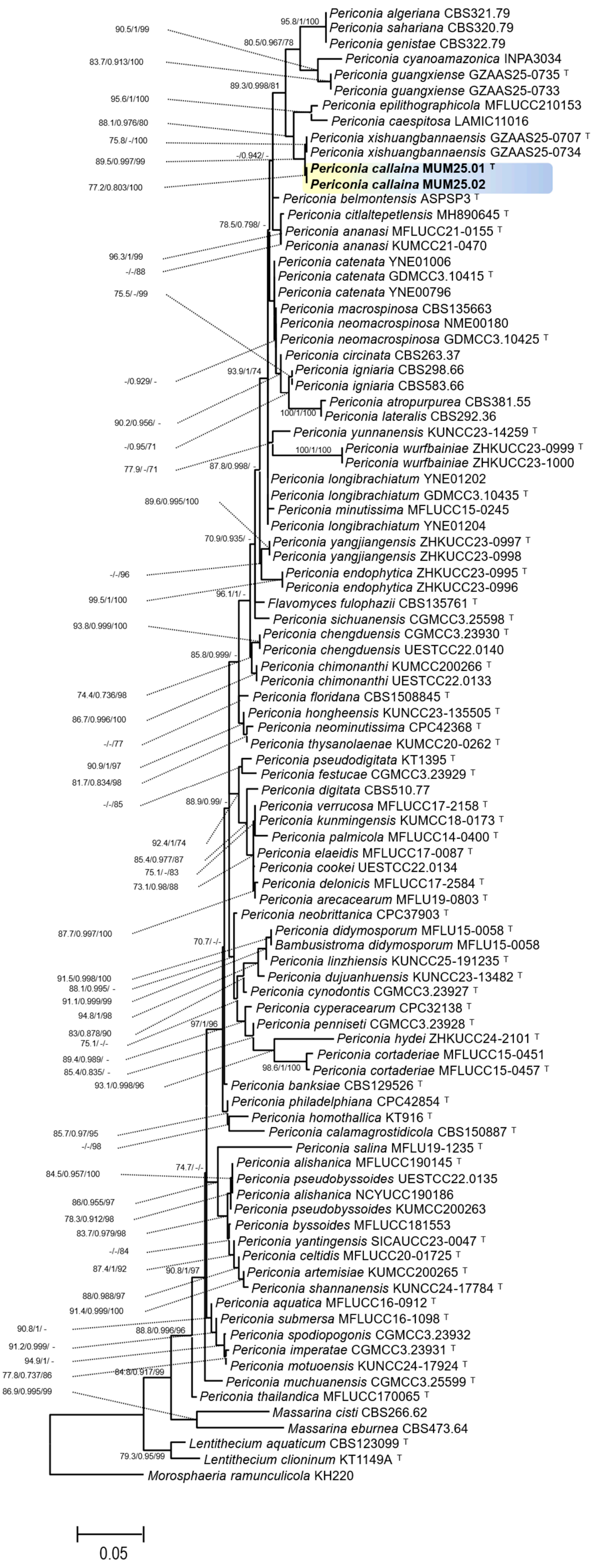

Supplementary Figure S1. Maximum likelihood (ML) tree based on sequence data from LSU showing the placement of the new species, *Periconia callaina*, described in this study. The scale bar indicates the number of substitutions per site. Nodes are labelled with SH-aLRT / aBayes / ultrafast bootstrap support values ≥ 70 % / 0.70 / 70 %, with type strains designated by superscript T. The novel species is highlighted in bold font. The tree is rooted with *Morosphaeria ramunculicola* (KH220).

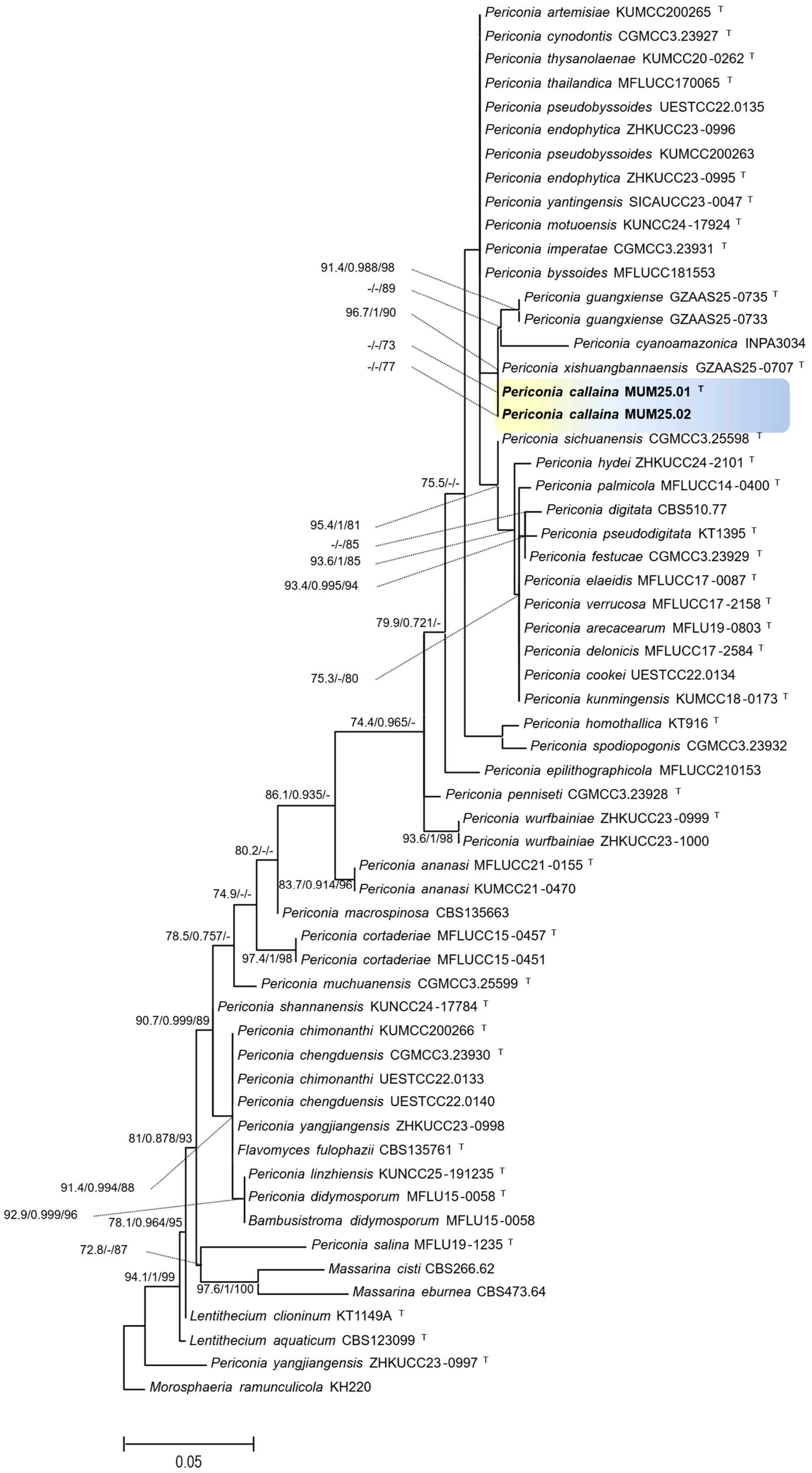

Supplementary Figure S3. Maximum likelihood (ML) tree based on sequence data from SSU showing the placement of the new species, *Periconia callaina*, described in this study. The scale bar indicates the number of substitutions per site. Nodes are labelled with SH-aLRT / aBayes / ultrafast bootstrap support values ≥ 70 % / 0.70 / 70 %, with type strains designated by superscript T. The novel species is highlighted in bold font. The tree is rooted with *Morosphaeria ramunculicola* (KH220).

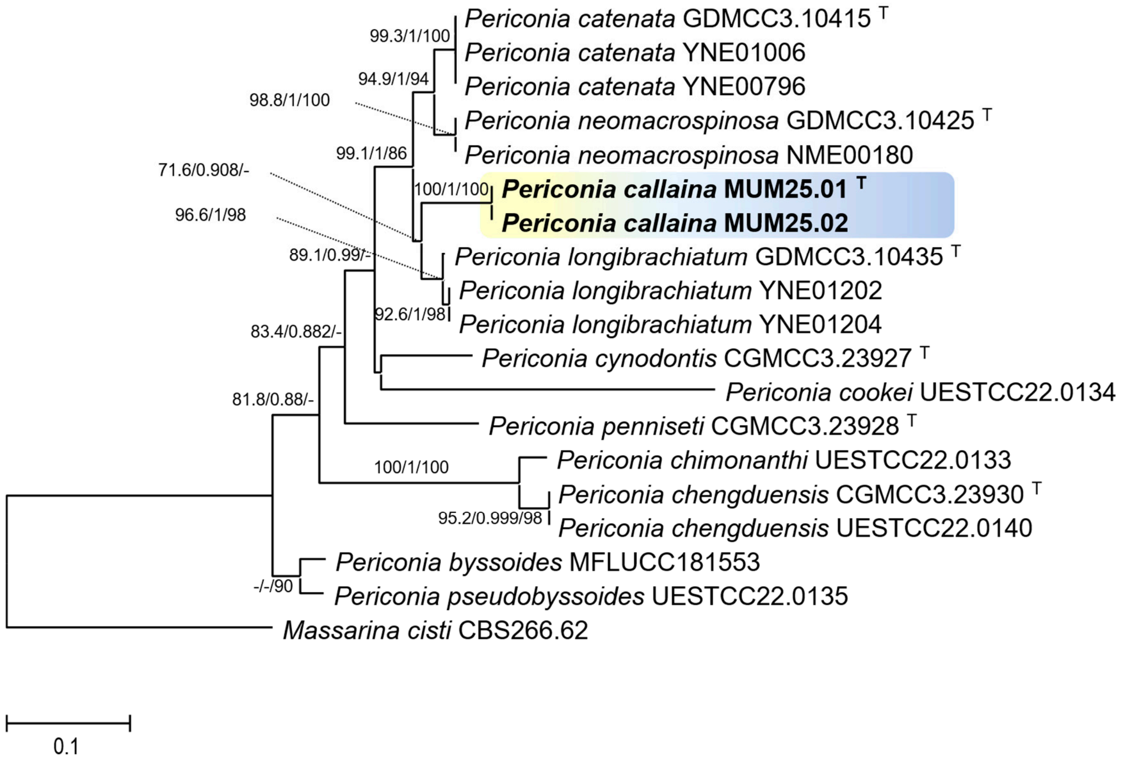

Supplementary Figure S4. Maximum likelihood (ML) tree based on sequence data from *RPB2* showing the placement of the new species, *Periconia callaina*, described in this study. The scale bar indicates the number of substitutions per site. Nodes are labelled with SH-aLRT / aBayes / ultrafast bootstrap support values ≥ 70 % / 0.70 / 70 %, with type strains designated by superscript T. The novel species is highlighted in bold font. The tree is rooted with *Massarina cisti* (CBS266.62).

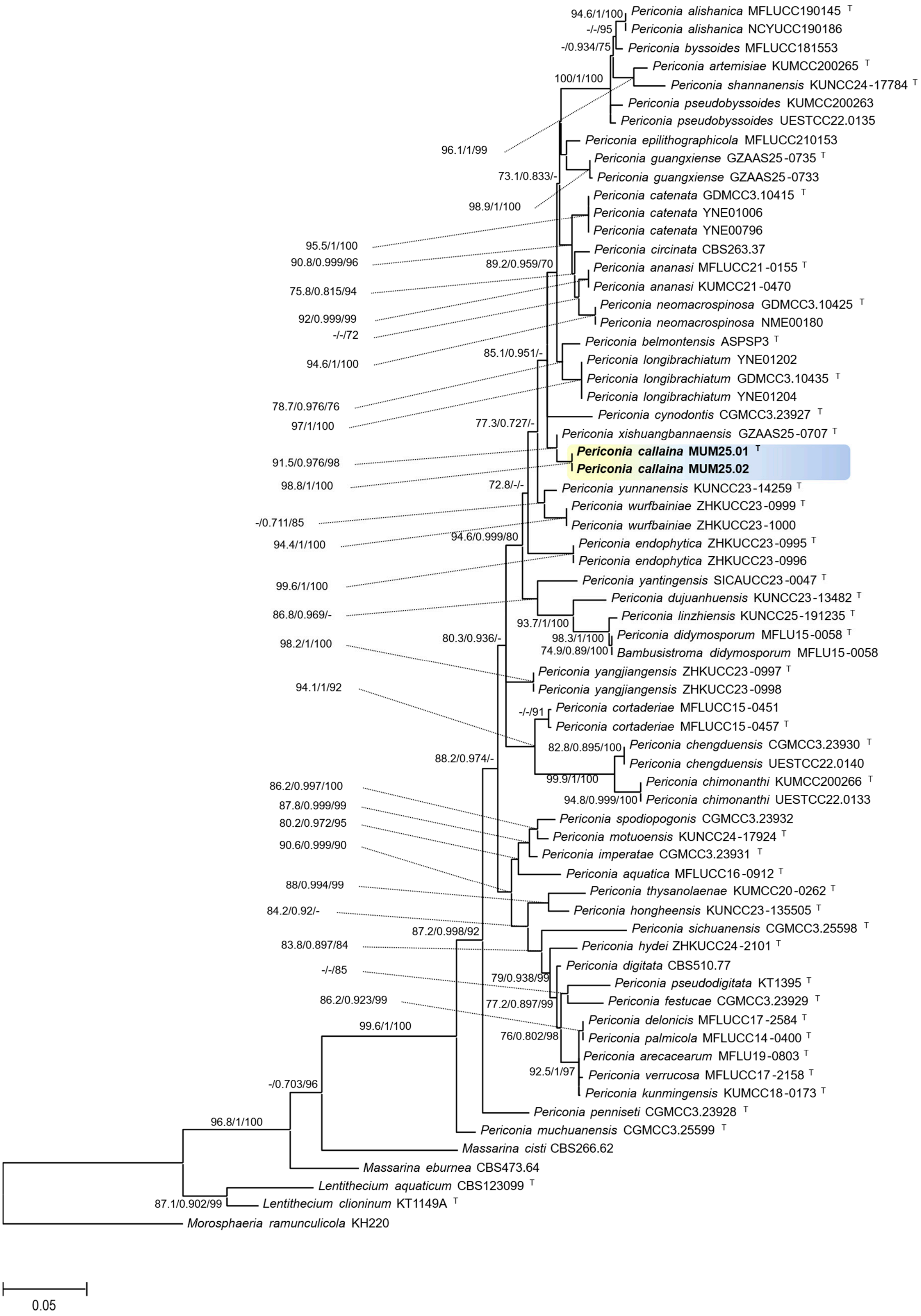

Supplementary Figure S5. Maximum likelihood (ML) tree based on sequence data from *TEF1α* showing the placement of the new species, *Periconia callaina*, described in this study. The scale bar indicates the number of substitutions per site. Nodes are labelled with SH-aLRT / aBayes / ultrafast bootstrap support values ≥ 70

% / 0.70 / 70 %, with type strains designated by superscript T. The novel species is highlighted in bold font. The tree is rooted with *Morosphaeria ramunculicola* (KH220).
