# Supplementary material for: Spores Among Feathers: Evaluating Fungal Diversity in Taxidermized Birds and the Discovery of a New Species
Source: Microorganisms. 2026 Aug 26;14(9):1894. doi: 10.3390/microorganisms14091894 (PMC13609718; doi:10.3390/microorganisms14091894)
Supplement: Supplementary file 1 [file microorganisms-14-01894-s001.zip › Supplementary materials - Tables S1-S3.pdf]

Supplementary Table S1 - Identification of all isolates retrieved in this study.

Isolate	Isolate ID	GenBank Accession Number [1]						
		ITS	LSU	SSU	*Btub*	*Tef1α*	*Rpb2*	*CaM*
Ext.37A		PV484568						PX434379
Ext.37B		PV484569						PX434380
Ext.37C		PV484570			PX434372			PX434381
Ext.37D		PV484571			PX434373			PX434382
Ext.37E		PV484572			PX434374			PX434383
Ext.37F		PV484573			PX434375			PX434384
Ext.37G		PV484574			PX434376			PX434385
Ext.37H		PV484575			PX434377			PX434386
Ext.41		PV484576	PV484675	PV484690				
Ext.42A		PV341358	PV387312	PV387571		PV423528	PV423526	
Ext.42B		PV341359	PV387313	PV387572		PV423529	PV423527	
Ext.43		PV484577	PV484676			PX434388		
Ext.44		PV484578	PV484677	PV484691				
Ext.45		PV484579	PV484678					
Ext.46		PV484580	PV484679					
Ext.47		PV484581	PV484680					
Ext.48		PV484582	PV484681		PX434378			
Ext.49		PV484583	PV484682					
Ext.50		PV484584		PV484692				
Ext.51		PV484585	PV484683					
Ext.52		PV484586	PV484684	PV484693				
Ext.53		PV484587	PV484685	PV484694				
Ext.54		PV484588					PX434387	
Ext.55		PV484589	PV484686					

Supplementary Table S2 - Strain collection references and GenBank accession numbers for the novel species outlined in this study, along with additional strains included in phylogenetic analyses, with *Massarina cisti* as the outgroup.

Species	Isolate	Origin	Host	GenBank accession no.				
				ITS	LSU	SSU	*Tef1α*	*Rpb2*
Periconia callaina **T**	**MUM–H 25.01**	**Portugal**	**Taxidermied bird**	**PV341358**	**PV387312**	**PV387571**	**PV423526**	**PV423528**
Periconia callaina	**MUM 25.02**	**Portugal**	**Taxidermied bird**	**PV341359**	**PV387313**	**PV387572**	**PV423527**	**PV423529**
Periconia algeriana	CBS 321.79	Algeria	*Genista saharae*, dead twig	MH861212	MH872979	–	–	–
Periconia alishanica T	MFLUCC 19–0145	Taiwan	*Ficus septica*	MW063165	MW063229	–	MW183790	–
Periconia alishanica	NCYUCC 19–0186	Taiwan	*Macaranga tanarius*	MW063166	MW063230	–	MW183791	–
Periconia ananasi T	MFLUCC 21-0155	Thailand	Pineapple, decaying leaves and stems	OL753685	OL606153	OL606142	OL912946	–
Periconia ananasi	KUMCC 21-0470	Thailand	Pineapple, dead leaves	OM102539	OL985955	OL979226	OM007977	–
Periconia aquatica T	MFLUCC 16-0912	China	Submerged wood	KY794701	KY794705	–	KY814760	–
Periconia artemisiae T	KUMCC 20–0265	China	*Artemisia apiacea*	MW448657	MW448571	MW448658	MW460898	–
Periconia atropurpurea	CBS 381.55	Mozambique	Soil	MH857524	MH869061	–	–	–
Periconia banksiae T	CBS 129526	Australia	*Banksia aemula*, leaves	–	NG_064279	–	–	–
Periconia byssoides	MFLUCC 18–1553	China	*Magnolia grandiflora*, plant	MK347806	MK348025	MK347914	MK360068	MK434858
Periconia caespitosa T	LAMIC 110 16	Brazil	Decaying leaves	MH051906	MH051907	–	–	–
Periconia chengduensis T	CGMCC 3.23930	China	*Cenchrus purpureus*, dead culms	OP955987	OP956012	OP956056	OP961453	OP961469
Periconia chengduensis	UESTCC 22.0140	China	*Miscanthus sinensis*, dead culms	OP955977	OP956002	OP956046	OP961443	OP961465
Periconia chimonanthi T	KUMCC 20–0266	China	*Chimonanthi praecox*	NR_176752	MW448572	MW448656	MW460897	–
Periconia chimonanthi	UESTCC 22.0133	China	*Arundo donax*, dead leaves	OP955964	OP955989	OP956033	OP961430	OP961455
Periconia circinata	CBS 263.37	United Kingdom	Soil	MW810265	MH867413	–	MW735660	–
Periconia citlaltepetlensis T	IOM 325319	Mexico	Volcanic Glacier	MH890645	MT625978	–	–	–
Periconia cookei	UESTCC 22.0134	China,	*Digitaria sanguinalis*, dead culms	OP955968	OP955993	OP956037	–	OP961459
Periconia cortaderiae	MFLUCC 15-0451	Thailand	*Cortaderia* sp, dead stems and leaves	KX965734	KX954403	KX986346	KY429208	–
Periconia cortaderiae T	MFLUCC 15-0457	Thailand	*Cortaderia* sp, dead stems and leaves	KX965732	KX954401	KX986345	KY310703	–

Periconia cynodontis T	CGMCC 3.23927	China	*Cynodon dactylon*, dead leaves	OP909925	OP909921	OP909920	OP961434	OP961460
Periconia cyperacearum T	CPC 32138	Australia	Plant leaves	NR_160357	NG_064549	–	–	–
Periconia delonicis T	MFLUCC 17-2584	Thailand	Plant	–	NG_068611	NG_065770	MK360071	–
Periconia didymosporum T	MFLUCC 15-0058	Thailand	Decaying culm of bamboo	KP761734	KP761731	KP761738	KP761728	–
Periconia digitata	CBS 510.77	Kuwait	Salt-marsh mud	LC014584	AB807561	AB797271	AB808537	–
Periconia elaeidis T	MFLUCC 17-0087	Thailand	*Elaeis guineensis*	MG742713	MH108552	MH108551	–	–
Periconia endophytica T	ZHKUCC 23-0995	China	*Wurfbainia villosa*, healthy leaf	OR995582	OR995588	PP277722	PP025968	–
Periconia endophytica	ZHKUCC 23-0996	China	*Wurfbainia villosa*, healthy leaf	OR995583	OR995589	PP277723	PP025969	–
Periconia epilithographicola T	CBS 144017	Costa Rica	19th century art lamina	NR_157477	–	–	–	–
Periconia epilithographicola	MFLUCC 21–0153	Thailand	Pineapple, decaying leaves and stems	OL753687	OL606155	OL606144	OL912948	–
Periconia festucae T	CGMCC 3.23929	China	*Festuca elata*, dead culms	OP955973	OP955998	OP956042	OP961439	–
Periconia genistae	CBS 322.79	Algeria	*Genista saharae*, dead twig	MH861213	MH872980	–	–	–
Periconia homothallica T	KT 916	Japan	*Phragmites japonica*	AB809645	AB807565	AB797275	–	–
Periconia igniaria	CBS 298.66	South Africa	*Acacia karroo*, leaf litter	MH858798	MH870438	–	–	–
Periconia igniaria	CBS 583.66	South Africa	*Acacia karroo*, leaf litter	MH858888	MH870553	–	–	–
Periconia imperatae T	CGMCC 3.23931	China	*Imperata cylindrica*, dead leaves and culms	OP955984	OP956009	OP956053	OP961450	–
Periconia kunmingensis T	KUMCC 18-0173	China	Dead hanging branches of fern	MH892346	MH892399	OR225814	MH908963	–
Periconia lateralis	CBS 292.36	USA	Hospital air	MH855804	MH867311	–	–	–
Periconia macrospinosa	CBS 135663	Hungary	*Festuca vaginata*, roots	KP183999	KP184038	KP184080	–	–
Periconia minutissima	MFLUCC 15-0245	China	Submerged wood	KY794703	KY794707	–	–	–
Periconia neobrittanica T	CPC 37903	USA	*Melaleuca styphelioides x lanceolata*, leaves	NR_166344	NG_068342	–	–	–
Periconia neominutissima T	CPC 42368	France	*Poaceae*, leaves	OQ628478	OQ629060	–	–	–
Periconia palmicola T	MFLUCC 14-0400	Thailand	Dead branch of herb	–	NG_068917	MN648319	MN821070	–
Periconia penniseti T	CGMCC 3.23928	China	*Pennisetum*, dead culms	OP955971	OP955996	OP956040	OP961437	OP961462
Periconia philadelphiana T	CPC 42854	USA	HVAC coil swab	OQ628486	OQ629068	–	–	–
Periconia prolifica	DBOF74	India	Marine sediments	JQ724435	–	–	–	–
Periconia pseudobyssoides	KUMCC 20–0263	China	*Scrophularia ningpoensis*	MW444851	MW444852	MW444853	MW460894	–
Periconia pseudobyssoides	UESTCC 22.0135	China	*Digitaria sanguinalis*, dead leaves	OP955965	OP955990	OP956034	OP961431	OP961456
Periconia pseudodigitata T	KT 1395	Japan	*Phragmites australis*	NR_153490	NG_059396	NG_064850	AB808540	–

Periconia sahariana T	CBS 320.79	Algeria	*Genista saharae*, dead twig	MH861211	MH872978	–	–	–
Periconia salina T	MFLU 19-1235	Wales	Unidentified bark, buried in sand dune	MN047086	MN017846	MN017912	–	–
Periconia spodiopogonis	CGMCC 3.23932	China	*Spodiopogon* sp., dead culms	OP955963	OP955988	OP956032	OP961429	–
Periconia submersa T	MFLUCC 16-1098	China	Submerged wood	KY794702	KY794706	–	–	–
Periconia thailandica T	MFLUCC 17–0065	Thailand	Decaying bamboo	KY753887	KY753888	KY753889	–	–
Periconia thysanolaenae T	KUMCC 20-0262	China	*Thysanolaena latifolia*	MW442967	MW444850	MW448659	MW460896	–
Periconia variicolor T	CBS 120374	Puerto Rico	Salt pond water	DQ336713	–	–	–	–
Periconia verrucosa T	MFLUCC 17-2158	Belgium	*Clematis viticella*	MT310617	MT214572	MT226686	MT394631	–
Periconia wurfbainiae T	ZHKUCC 23-0999	China	*Wurfbainia villosa*, dead stem	OR995586	OR995592	PP277726	PP025972	–
Periconia wurfbainiae	ZHKUCC 23-1000	China	*Wurfbainia villosa*, dead stem	OR995587	OR995593	PP277727	PP025973	–
Periconia yangjiangensis T	ZHKUCC 23-0997	China	*Wurfbainia villosa*, dead stem	OR995584	OR995590	PP277724	PP025970	–
Periconia yangjiangensis	ZHKUCC 23-0998	China	*Wurfbainia villosa*, dead stem	OR995585	OR995591	PP277725	PP025971	–
Periconia cyanoamazonica T	INPA 3034	Brazil	Lab contaminant	PP572457	PP572458	PP572459	–	–
Periconia guangxiense T	GZAAS 25-0735	China	Decaying submerged wood	PX118886	PX118882	PX118890	PX522310	–
Periconia guangxiense	GZAAS 25-0733	China	Decaying submerged wood	PX118887	PX118883	PX118891	PX522311	–
Periconia xishuangbannaensis T	GZAAS25-0707	China	Zea mays, dead leaves	PX118888	PX118884	PX118892	PX522312	–
Periconia xishuangbannaensis	GZAAS25-0734	China	Zea mays, dead leaves	PX118889	PX118885	–	–	–
Periconia catenata T	GDMCC 3.10415	China	Capillipedium sp., roots	PP106201	PP106225	–	PP112539	PP112512
Periconia catenata	YNE00796	China	Capillipedium sp., roots	PP106200	PP106223	–	PP112538	PP112510
Periconia catenata	YNE01006	China	Capillipedium sp., roots	PP106202	PP106226	–	PP112540	PP112511
Periconia neomacrospinosa T	GDMCC 3.10425	China	Cleistogenes serotina, roots	PP106192	PP106212	–	PP112530	PP112502
Periconia neomacrospinosa	NME00180	China	Cleistogenes serotina, roots	PP106191	PP106211	–	PP112529	PP112501
Periconia longibrachiatum T	GDMCC 3.10435	China	Poaceae sp., roots	PP106205	PP106231	–	PP112543	PP112515
Periconia longibrachiatum	YNE01202	China	Poaceae sp., roots	PP106203	PP106228	–	PP112541	PP112513
Periconia longibrachiatum	YNE01204	China	Poaceae sp., roots	PP106204	PP106229	–	PP112542	PP112514
Periconia belmontensis T	URM 9266	Brazil	Seawater	PQ248713.1	PX600748	–	PX640513	–
Periconia motuoensis T	KUNCC24-17924	China	Poaceae sp., dead sheaths	PQ373177	PQ438054	PQ438116	PQ661220	–
Periconia linzhiensis T	KUNCC25-191235	China	Bamboo	PQ684989	PQ675408	PQ675368	PQ671465	–
Periconia arecacearum T	MFLU 19-0803	Thailand	*Cocos nucifera*	PP592462	PP621090	PP639222	PP828795	–
Periconia calamagrostidicola T	CBS 150887	Netherlands	*Calamagrostis arenaria*, old leaves	PP791432	P791460	–	–	–

Periconia celtidis T	MFLUCC 20-01725	China	*Celtis tetrandra*, dead leaves	NR_174830	NG_079543	–	–	–
Periconia dujuanhuensis T	KUNCC 23-13482	China	Submerged decaying wood	PQ340469	PP189907	–	PQ456957	–
Periconia floridana T	CBS 1508845	USA	Outside air	NR_197917	NG_244042	–	–	–
Periconia hongheensis T	KUNCC 23-13550	China	Submerged decaying wood	PQ340467	PP189905	–	PQ456958	–
Periconia hydei T	ZHKUCC 24-2101	China	*Stipa capillata*, submerged decaying twigs	PV434171	PV436640	PV437586	PV588047	–
Periconia muchuanensis T	CGMCC 3.25599	China	Bamboo, dead branches	PQ067868	PQ067698	PQ066546	PQ278553	–
Periconia shannanensis T	KUNCC24-17784	China	Dead Artemisia	PP968552	PP968555	PP968558	PQ226770	–
Periconia sichuanensis T	CGMCC 3.25598	China	Bamboo, dead branches	PQ067867	PQ067697	PQ066545	PQ278551	–
Periconia yantingensis T	SICAUCC 23-0047	China	*Juglans regia*, dead twigs	PP060663	PP057956	PP003824	PP061142	–
Periconia yunnanensis T	KUNCC 23-14259	China	Submerged decaying wood	PQ340468	PP189906	–	PQ456960	–
Bambusistroma didymosporum	MFLU 15-0058	–	–	KP761734	KP761731	KP761738	KP761728	–
Flavomyces fulophazii T	CBS 135761	Hungary	*Festuca vaginata, roots*	NR_137960	NG_058131	NG_061191	–	–
Lentithecium aquaticum T	CBS 123099	–	–	NR_160229	NG_064211	NG_016507	GU349068	–
Lentithecium clioninum T	KT1149A	Japan	Dead wood	LC014566	AB807540	AB797250	AB808515	–
Massarina cisti	CBS 266.62	France	*Cistus albidus*	LC014568	AB807539	FJ795490	AB808514	FJ795464
Massarina eburnea	CBS473.64	–	–	–	GU301840	GU296170	GU349040	–
Morosphaeria ramunculicola	KH220	Japan	*Rhizophora mucronata*	–	AB807554	AB797264	AB808530	–

GenBank accession numbers, with the newly generated sequences indicated in bold. Outgroup highlighted in grey.

Type strains are designated with T.

Culture Collection designations: CBS – CBS-KNAW Culture Collection, Westerdijk Fungal Biodiversity Institute, Utrecht, Netherlands; **CGMCC –** China General Microbiological Culture Collection Center Beijing, China; **CPC –** Culture Collection of Pedro Crous, Netherlands; **DBOF –** DNA Barcoding Ocean Fungi; **GDMCC –** Guangdong Microbial Culture Collection Center, Guangdong Institute of Microbiology, Guangzhou, China; **GZAAS –** Guizhou Academy of Agriculture Sciences Herbarium, Guizhou, China; **INPA –** Instituto Nacional de Pesquisas da Amazônia, Manaus, Brazil; **IOM –** Instituto de Oftalmología "Fundación Conde de Valenciana" IAP Mexico Culture Collection; **KT –** Kaz Tanaka; **KUMCC –** Kunming Institute of Botany Culture Collection, Kunming, China; **LAMIC –** Laboratorio de Micologia, Universidade Estadual de Feira de Santana, Brazil; **MFLUCC –** Mae Fah Luang University Culture Collection,

Chiang Rai, Thailand; **NCYUCC –** National Chiayi University Culture Collection, Taiwan, China; **MUM –** Micoteca da Universidade do Minho, Centre of Biological Engineering, University of Minho, Minho, Portugal; **RUTPP –** Rutgers Mycological Herbarium, New Brunswick, New Jersey, USA; **SICAUC** – Sichuan Agricultural University Culture Collection, Sichuan, China; **TNS-F –** NationalMuseum of Nature and Science, Department of Botany, Tsukuba, Japan; **UESTCC –** University of Electronic Science and Technology Culture Collection, Chengdu, China; **URM** – University Recife Mycology, Universidade Federal de Pernambuco, Recife, Brazil; **ZHKUCC –** Mycological Herbarium of Zhongkai University of Agriculture and Engineering, Guangzhou, China.

Supplementary Table S3 - The best–fit substitution models for each partition determined by ModelFinder based on the Akaike Information Criterion.

Species	Genetic markers	No. sequences	Alignment length (bp)	No. unique site patterns	No. Parsimony–Informative Sites	No. invariant sites	No. constant sites	Substitution Model
Periconia callainae	ITS	93	496	276	195	239	239	TIM2+F+G4
	LSU	95	774	224	125	573	573	TNe+I+G4
	SSU	59	1039	146	55	842	842	K2P+I+G4
	Rpb2	19	879	300	227	559	559	TIM3e+G4
	Tef1α	67	668	182	141	490	490	GTR+F+I+G4
	Concatenated	99	3856	1127	743	2703	2703	TIM2+F+I+G4
